# Genetic and clinical characteristics of treatment-resistant depression using primary care records in two UK cohorts

**DOI:** 10.1038/s41380-021-01062-9

**Published:** 2021-03-22

**Authors:** Chiara Fabbri, Saskia P. Hagenaars, Catherine John, Alexander T. Williams, Nick Shrine, Louise Moles, Ken B. Hanscombe, Alessandro Serretti, David J. Shepherd, Robert C. Free, Louise V. Wain, Martin D. Tobin, Cathryn M. Lewis

**Affiliations:** 1 Social, Genetic and Developmental Psychiatry Centre, Institute of Psychiatry, Psychology and Neuroscience, King’s College London, London, UK; 2 Department of Biomedical and Neuromotor Sciences, University of Bologna, Bologna, Italy; 3 Department of Health Sciences, University of Leicester, Leicester, UK; 4 NIHR Leicester Biomedical Research Centre, University of Leicester, Leicester, UK; 5 Department of Respiratory Sciences, University of Leicester, Leicester, UK; 6 Department of Medical and Molecular Genetics, Faculty of Life Sciences and Medicine, King’s College London, London, UK

**Keywords:** Depression, Genetics

## Abstract

Treatment-resistant depression (TRD) is a major contributor to the disability caused by major depressive disorder (MDD). Primary care electronic health records provide an easily accessible approach to investigate TRD clinical and genetic characteristics. MDD defined from primary care records in UK Biobank (UKB) and EXCEED studies was compared with other measures of depression and tested for association with MDD polygenic risk score (PRS). Using prescribing records, TRD was defined from at least two switches between antidepressant drugs, each prescribed for at least 6 weeks. Clinical-demographic characteristics, SNP-based heritability (*h*^*2*^_*SNP*_) and genetic overlap with psychiatric and non-psychiatric traits were compared in TRD and non-TRD MDD cases. In 230,096 and 8926 UKB and EXCEED participants with primary care data, respectively, the prevalence of MDD was 8.7% and 14.2%, of which 13.2% and 13.5% was TRD, respectively. In both cohorts, MDD defined from primary care records was strongly associated with MDD PRS, and in UKB it showed overlap of 71–88% with other MDD definitions. In UKB, TRD vs healthy controls and non-TRD vs healthy controls *h*^*2*^_*SNP*_ was comparable (0.25 [SE = 0.04] and 0.19 [SE = 0.02], respectively). TRD vs non-TRD was positively associated with the PRS of attention deficit hyperactivity disorder, with lower socio-economic status, obesity, higher neuroticism and other unfavourable clinical characteristics. This study demonstrated that MDD and TRD can be reliably defined using primary care records and provides the first large scale population assessment of the genetic, clinical and demographic characteristics of TRD.

## Introduction

Major depressive disorder (MDD) is a common psychiatric disorder affecting more than 264 million people worldwide and it is the fourth-leading cause of disability [1].

The study of factors determining the course of MDD and response to treatments has been a major research area, with the aim of providing better instruments for personalised health care and facilitate recovery, i.e., the return to the pre-morbid level of health and functioning, a condition associated with a reduced risk of depressive relapse [2]. A substantial proportion of patients with MDD do not reach remission, even after multiple antidepressant treatments [3]. Antidepressants are a first line treatment for MDD, with over 40 compounds currently available. A network meta-analysis showed a clear benefit of antidepressants over placebo with some differences between drugs, but there are large inter-individual differences in response [4]. In the United Kingdom, MDD is usually treated in primary care and antidepressant treatment is recommended for moderate to severe depression [5]. Therefore, the study of modulators of response to antidepressants in primary care represents a fundamental step to improve the health care of patients with MDD.

Treatment-resistant depression (TRD), usually defined as lack of response to at least two antidepressants, has a prevalence of 7% in MDD cases in Scottish primary care (based on Electronic Health Records [EHR]) [6] and 22% in Canadian primary care (based on a questionnaire completed by physicians) [3]. TRD is associated with social and occupational impairment, suicidal thoughts, decline of physical health, increased health care utilization and higher all-cause mortality compared with non-TRD [7, 8]. Recently, difficult to treat depression (DTD) has been proposed as an alternative way of conceptualizing the issue of poor response to multiple treatments in MDD. The term DTD aims to capture the complexity of illness management in these individuals and the view that MDD is treatable (“difficult”, not “impossible”) [9]. To align with previous studies, we did not use DTD as terminology in this work, but we acknowledge that DTD should be considered as an alternative term [6, 10–12].

Symptom remission during antidepressant treatment is partly influenced by genetic factors, with an SNP-based heritability (*h*^*2*^_*SNP*_) of 0.132 (SE = 0.056) [13]. Three genome-wide association studies (GWAS) have identified no genetic variants associated with TRD [6, 11, 10], while the most recent 23andMe GWAS identified one genomic region in 10p11.1, spanning multiple genes and having rs150245813 as lead SNP [12]. This GWAS estimated both the *h*^*2*^_*SNP*_ of TRD vs non-TRD (0.08, SE = 0.04, in the meta-analysis) and *h*^*2*^_*SNP*_ of TRD vs healthy controls (0.17 [SE = 0.05] and 0.19 [SE = 0.04], depending on the sample). Few studies have had sufficient power to investigate the genetic overlap of TRD with other psychiatric traits and the available evidence is very limited [6]. No studies of genetic correlation with non-psychiatric traits have been reported.

Current studies in TRD have been hampered by small sample sizes and low power, but EHR provide an exciting opportunity for large scale pharmacogenetic studies at low cost and with high classification accuracy [14]. Clinical trials of sufficient size for well-powered pharmacogenetic analysis of antidepressant response are not available, therefore EHR and digital phenotyping appear the most promising strategy [15]. In this study, we used primary care EHR linked to the Extended Cohort for E-health, Environment and DNA (EXCEED) cohort to develop and test an algorithm to define MDD and TRD (these data were available before similar data in UK Biobank (UKB) and extensively annotated); then, this was applied to UKB to validate and extend the results to a larger cohort. Specifically, using primary care data, we:

1. Identified patients with MDD, validated our algorithm in EXCEED and UKB by assessing the genetic overlap with a large, independent MDD sample [16], and by phenotypic comparisons with other MDD definitions available in UKB;
2. Identified patients with TRD, then studied their clinical and socio-demographic features compared with non-TRD (both cohorts), calculating TRD *h*^*2*^_*SNP*_ and assessing the genetic correlation of TRD with other psychiatric and non-psychiatric traits (UKB).

## Materials and methods

### EXCEED and UK Biobank cohorts

EXCEED is a prospective cohort of over 10,000 individuals from Leicester, Leicestershire and Rutland with genetic data and linkage to primary care and hospital EHR. Primary care data is available for 8,926 participants, with date and clinical code (Read v2 or CTV3) [17].

UKB is a prospective population-based study of ~500,000 individuals recruited across the United Kingdom. To date, primary care data have been obtained for ~230,000 participants. Clinical (Read v2 or CTV3) and drug codes (Read v2, BNF 2 and/or dm+d) and associated dates are available for primary care events [18]. Further information is available in Supplementary Methods.

Genome-wide genotyping is available in over 60% of the EXCEED cohort and all UKB participants; details on the arrays used, quality control and imputation are in Supplementary Methods.

Both studies received ethnical approval and participants provided written informed consent. We did not have permission to match individuals in EXCEED and UKB to check for overlap between the two samples. However, there was no UKB assessment centre in Leicester and the closest one (Nottingham) was over 15 miles from all the GP practices which recruited for EXCEED, reasonably excluding any overlap since individuals were invited to take part in UKB when they lived within a radius of 10 miles from an assessment centre [19].

### Definition of MDD and TRD

In both cohorts, participants with MDD were identified as those having:

- At least two diagnostic codes for a depressive disorder, at any time point (two codes were required to reduce the risk of miscoding and inclusion of individuals with other psychiatric disorders as main diagnosis);
- No diagnostic code for bipolar disorders (to exclude cases with bipolar depression), psychotic disorders or substance use-related disorders, as these depression cases are reported to show different clinical-demographic characteristics, response to treatments and pathogenetic mechanisms [20–22].

**Fig. 1:**
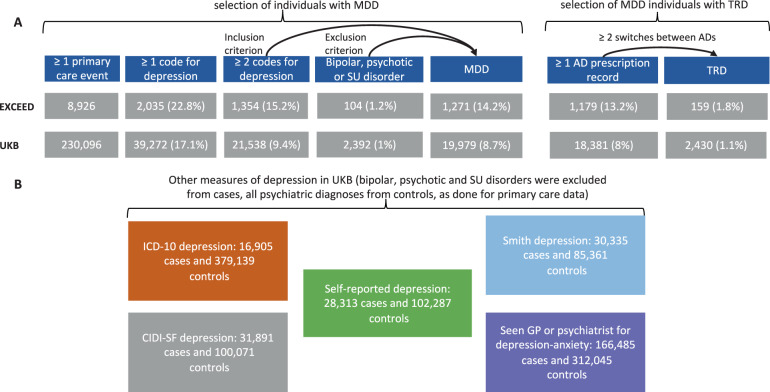
Definition of MDD and TRD in both cohorts. Selection of individuals with depression and treatment-resistant depression (TRD) in EXCEED and UKB primary care data (**A**) and other measures of depression used as comparison in UKB (**B**). MDD major depressive disorder, SU substance use, AD antidepressant.

Participants with TRD were defined as those with MDD having at least two switches between different antidepressant drugs (independently from the class) satisfying the following criteria (Fig. 1):

- Each drug was prescribed for at least 6 consecutive weeks (noting that adequate duration for efficacy is 4 weeks, and our conservative threshold should reduce the risk that drug switch was due to side effects [6]);
- The time interval between the prescription of two consecutive drugs was no longer than 14 weeks (to ensure that treatment had not been suspended).

To evaluate a proxy of poor compliance to treatment as a possible contributor to the poorer response observed in TRD, for each subject we calculated the proportion of adequate prescription intervals, defined by 14 weeks or less between subsequent antidepressant prescriptions. This was not circular with TRD definition, since in this case all time intervals between subsequent prescriptions (same or different antidepressant) were considered and not only the time interval between different drugs when switching.

The R code used to produce these phenotypes in UKB is available at: https://github.com/chiarafabbri/MDD_TRD_study.

### Statistical analyses

#### Phenotypic analyses

To validate the definition of MDD using primary care EHR, we cross-classified with five UKB depression phenotypes (Fig. 1): (1) the one defined by the Composite International Diagnostic Interview Short Form (CIDI-SF), which was part of the Mental Health Questionnaire (MHQ) [23], (2) hospital diagnosis (ICD-10 codes F32-F33-F34.1) (3) self-reported depression diagnosed by a professional (4) help-seeking for depression and (5) Smith et al. definition [24]. We excluded individuals with bipolar, psychotic or substance use disorders from cases and any psychiatric diagnosis from controls; further details are in the Supplementary Methods.

In both EXCEED and UKB, TRD can only be defined using primary care EHR, so we assessed the clinical and socio-demographic characteristics of TRD and non-TRD cases and we compared these findings with the existing literature on TRD in order to validate our TRD phenotype. These analyses were adjusted for possible confounding factors. We also tested if antidepressant combinations or augmentation with an antipsychotic or mood stabilizer for >30 days were more common in TRD than non-TRD, as these strategies are recommended for TRD [25].

We checked if the missingness proportion in clinical-demographic variables was similar between TRD and non-TRD groups in order to identify differences that may influence the results.

In UKB we analysed and/or described additional variables compared to EXCEED because: (1) UKB included primary care data collected across England, Wales and Scotland [18] rather than an area of England (EXCEED [17]), therefore it may be more representative of UK primary care practices than EXCEED; (2) UKB has additional phenotypic data [23].

#### Genetic analyses

We calculated polygenic risk scores (PRS) for MDD, schizophrenia and bipolar disorder (Supplementary Table 1) to test their association with MDD in primary care and other depression phenotypes described above. We hypothesized that prediction would be stronger for MDD PRS than other psychiatric disorders. For each of the considered depression phenotypes, participants with no psychiatric disorder according to that specific measure were considered as healthy controls (Fig. 1).

PRS were calculated using PRSice v.2 [26] and genotyped variants at 11 *p* value thresholds (*P*_*T*_) and the most predictive *P*_*T*_ was selected (see Supplementary Methods). Logistic regression models were used to estimate associations between the phenotype and each PRS adjusting for six genetic ancestry principal components, assessment centre and batch effects in UKB and six genetic ancestry principal components and primary care practice in EXCEED. The proportion of variance explained by PRS on the liability scale [27] was estimated assuming MDD prevalence of 10.8% for case-control comparisons [28]. A Bonferroni correction was applied considering the number of traits and *P*_*T*_ tested (nominal *p* values are reported, the alpha threshold is reported in the results tables).

For the estimation of SNP-based heritability (*h*^*2*^_*SNP*_) of TRD and non-TRD in UKB (these analyses would not have adequate power in EXCEED), we compared the results using three methods: genome-wide complex trait analysis (GCTA) [29], Genome-wide Complex Trait Bayesian (GCTB) [30] and linkage disequilibrium score regression (LDSC) [31] (see Supplementary Methods; the used GCTA and GCTB code is available at https://github.com/chiarafabbri/MDD_TRD_study). A set of 11,188 healthy controls (no psychiatric disorder) was selected after a power estimation (Supplementary Methods). *h*^*2*^_*SNP*_ was transformed to the liability scale using a range of possible population prevalences [3, 28, 32]. We evaluated the possibility that *h*^*2*^_*SNP*_ estimates may be inflated by selecting extremes of the controls (individuals without any psychiatric disorder) and cases (individuals with ≥ two diagnostic records of depression) distributions [32]. This was performed by comparing *h*^2^_*SNP*_ obtained using a set of controls of the same size without screening for psychiatric disorders other than MDD and by considering the prevalence of having at least one code for depression instead of two as corresponding to the population prevalence of MDD from the literature [32].

Genetic correlations (*r*_*g*_) with selected psychiatric and non-psychiatric traits were estimated using LDSC [31] (Supplementary Table 2). Three GWAS were performed: TRD vs non-TRD, TRD vs healthy controls and non-TRD vs healthy controls, using BGENIE v1.2 and imputed genotype dosages; [33] phenotypes were residualised for six genetic ancestry principal components, assessment centre and batch effects. *R*_*g*_ estimates, previous studies on TRD and a power analysis (R package “avengeme”) were used to guide the selection of PRS tested for association with TRD vs non-TRD [6] (Supplementary Table 1 and Supplementary Methods). Bonferroni correction was applied.

## Results

In EXCEED and UKB, 8926 and 230,096 participants had at least one primary care event, and the prevalence of MDD was 14.24% (*n* = 1271) and 8.68% (*n* = 19,979), respectively. Among individuals with MDD with at least one record of antidepressant prescription, the prevalence of TRD was 13.49% (*n* = 159) and 13.2% (*n* = 2430) in EXCEED and UKB, respectively (Fig. 1).

In UKB we reported trends of depression diagnoses and antidepressant prescriptions (drugs and classes) over time (Supplementary Fig. 1).

### Validation of primary care diagnosed MDD

#### PRS of MDD, schizophrenia and bipolar disorder

**Fig. 2:**
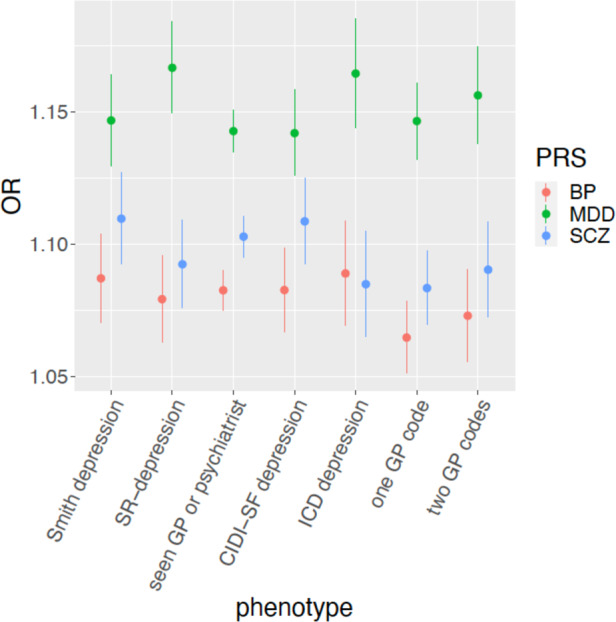
PRS of psychiatric disorders and risk of depression in UKB. Association between polygenic risk scores (PRS) for psychiatric disorders and depression phenotypes in UKB, showing odds ratios (OR) and 95% confidence intervals (PRS were standardized). SR-depression self-reported depression diagnosed by a professional, CIDI-SF Composite International Diagnostic Interview Short Form, GP general practitioner, BP bipolar disorder, MDD major depressive disorder, SCZ schizophrenia.

In the EXCEED and UKB cohorts, 557 and 17,807 participants with MDD had genetic data after quality control, and 2181 and 130,252 controls with no psychiatric diagnosis, respectively. MDD PRS was associated with primary care-defined MDD diagnosis (*p* = 6.05e–6 and *p* = 1.89e–71 in EXCEED and UKB, respectively; Supplementary Table 3), with a similar effect size in the two cohorts (z test comparing the effect size in the two samples: *z* = 1.58, *p* = 0.11). In EXCEED, schizophrenia and bipolar disorder PRS was not associated with MDD case-control status (Supplementary Table 3), while in UKB they had a significant effect that however was smaller than the effect of MDD PRS (z test to compare MDD PRS with schizophrenia and bipolar disorder PRS: *z* = 5.30, *p* = 1.14e–7, and *z* = 7.07, *p* = 1.54e–12, respectively; note that the association with schizophrenia PRS was not significantly stronger than the association with bipolar disorder PRS). The Nagelkerke *R*^*2*^ (liability scale) of MDD PRS was 1.2% and 0.6% in EXCEED and UKB, respectively. Given the larger sample size and greater generalizability of results, in UKB we compared the association of MDD PRS with depression defined using at least one vs at least two diagnostic codes for depression and found no difference (z test to compare the effect sizes: *z* = 1, *p* = 0.32). Both these definitions showed similar associations with MDD PRS when compared to other measures of depression in UKB (Fig. 2, Supplementary Table 3).

#### Comparison with other depression measures in UKB

**Fig. 3:**
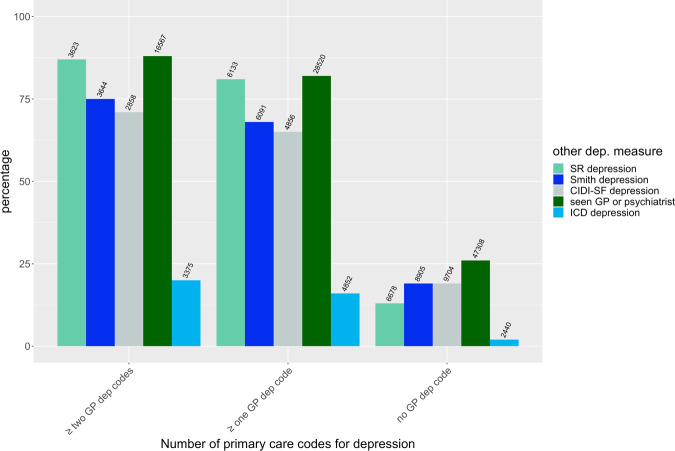
Overlap among different measures of depression in UKB. Percentage of UK Biobank participants having at least one, at least two or zero depression codes in primary care data who endorsed other measures of depression (ICD depression based on hospital records, Smith depression, self-reported (SR) depression diagnosed by a professional, depression according to the Composite International Diagnostic Interview Short Form (CIDI-SF) and help-seeking depression based on having seen a general practitioner (GP) or psychiatrist for depression-anxiety, see Supplementary Methods). The number of overlapping subjects is reported on top of each bar. See Supplementary Table 4 for further details on these comparisons.

MDD defined from primary care data showed overlap with other measures of depression in 71–88% of cases in UKB; 20% of participants with MDD according to primary care records also received a diagnosis of depression in a hospital setting (ICD-10 codes). For all the considered measures except CIDI-SF-defined depression and self-reported depression, the overlap was significantly higher for MDD defined using at least two diagnostic codes for depression compared to MDD defined using at least one diagnostic code (Fig. 3; Supplementary Table 4).

### Clinical and socio-demographic characteristics of TRD

Participants with TRD differed from non-TRD for many clinical and sociodemographic characteristics, all indicating that TRD is a more severe and debilitating disorder (Supplementary Table 5 for EXCEED and UKB, including details on the covariates considered; Supplementary Figs. 2–4 for UKB). TRD vs non-TRD cases were younger at first depression record as well as at first antidepressant prescription record (though these do not necessarily correspond to age at onset and age at first antidepressant prescription, given the nature of the data); they lived in areas with higher social deprivation, and lower SES was confirmed by other variables in UKB (education and income, Supplementary Table 5). TRD individuals showed higher levels of neuroticism and perceived loneliness, despite reporting similar rates of living alone and frequency of visits from family/friends, more frequent irritability and mood swings, less frequent moderate physical activity in UKB (Supplementary Table 5). In both EXCEED and UKB, participants with TRD vs non-TRD had higher BMI and higher risk of being obese, but similar risk of type 2 diabetes and cardiovascular diseases after adjusting for potential confounders, though in UKB they reported a higher risk of longstanding disabilities/infirmities (OR = 2.26 [2.06–2.49]). Missingness in the clinical-demographic variables was not different between TRD and non-TRD in both EXCEED and UKB (Supplementary Table 6), with the only exception of frequency of moderate physical activity in UKB (higher missingness in TRD, *p* = 3.14e–4).

**Fig. 4:**
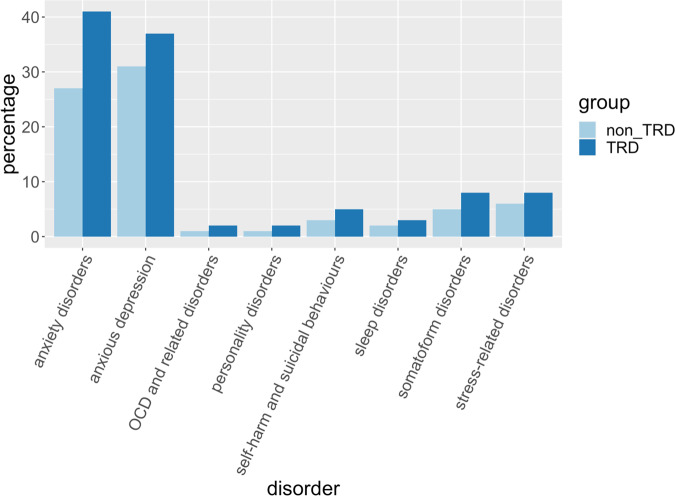
Psychiatric comorbidities in TRD and non-TRD in UKB. Psychiatric comorbidities in patients with treatment-resistant depression (TRD) and without TRD according to primary care records in UK Biobank. OCD = obsessive-compulsive disorder.

According to primary care EHR in UKB, patients with TRD had an increased risk of comorbidity with all the psychiatric disorders tested, particularly anxiety disorders (OR = 1.89 [1.73–2.07]), obsessive-compulsive disorder (OR = 3.03 [2.23–4.13]) and self-harm/suicidal behaviours (OR = 2.03 [1.67–2.48]); Fig. 4 and Supplementary Table 7. In UKB and EXCEED, antidepressant combinations were prescribed to 46 and 53% of TRD patients, respectively, and 8% of non-TRD subjects in both cohorts (OR = 5.66 [5.17–6.21] and OR = 7.68 [7.42–7.94], respectively). Differences in the type of antidepressant combinations and augmentation strategies were found in TRD vs non-TRD (UKB, Supplementary Fig. 5 and Supplementary Table 8).

In UKB, a higher proportion of prescription intervals of patients classified as TRD had adequate duration vs non-TRD, with a similar trend in EXCEED (Supplementary Table 5 and Supplementary Fig. 6). In both cohorts, participants with TRD showed a higher number of clinical and prescription records, particularly when considering records registered on different dates and their ratio with the number of years of available records (Supplementary Table 9 and Supplementary Fig. 7).

### SNP-based heritability of TRD in UKB

A total of 2146 TRD and 14,097 non-TRD participants were included after quality control (Supplementary Methods). The different methods used to estimate *h*^*2*^_*SNP*_ of TRD vs healthy controls and non-TRD vs healthy controls provided similar results (Table 1). *h*^*2*^_*SNP*_ (liability scale) of TRD and non-TRD were comparable, e.g., GCTB estimates were 0.25 [SE = 0.04] and 0.19 [SE = 0.02], respectively. The genetic correlation (LDSC) between TRD and non-TRD was 0.78 (SE = 0.08) and the *h*^*2*^_*SNP*_ of TRD vs non-TRD was 0.077 (SE = 0.027, *p* = 0.004) on the observed scale (case-only comparison).

**Table 1:** SNP-based heritability (SNP-h2) of treatment-resistant depression (TRD) and non-TRD compared with healthy controls in UKB.

Method	TRD SNP-h2			Non-TRD SNP-h2		
	Low prevalence *K* = 0.01	Estimated prevalence *K* = 0.023	High prevalence *K* = 0.03	Low prevalence *K* = 0.08	Estimated prevalence *K* = 0.085	High prevalence *K* = 0.10
GCTA	0.2660 (0.0412)	**0.3306 (0.0512)**	0.3581 (0.0555)	0.2495 (0.0238)	**0.2563 (0.0245)**	0.2765 (0.0264)
GCTB	0.1979 (0.0333)	**0.2459 (0.0414)**	0.2664 (0.0449)	0.1877 (0.0185)	**0.1928 (0.0190)**	0.2079 (0.0205)
LDSC	0.1748 (0.0271)	**0.2173 (0.0337)**	0.2354 (0.0365)	0.1877 (0.0174)	**0.1928 (0.0178)**	0.2080 (0.0193)

SNP-h2 was reported according to different possible values of prevalence (*K*) in the population; the most plausible values are reported in bold. *GCTA* genome-wide complex trait analysis, *GCTB* Genome-wide Complex Trait Bayesian, *LDSC* linkage-disequilibrium score regression.

**Fig. 5:**
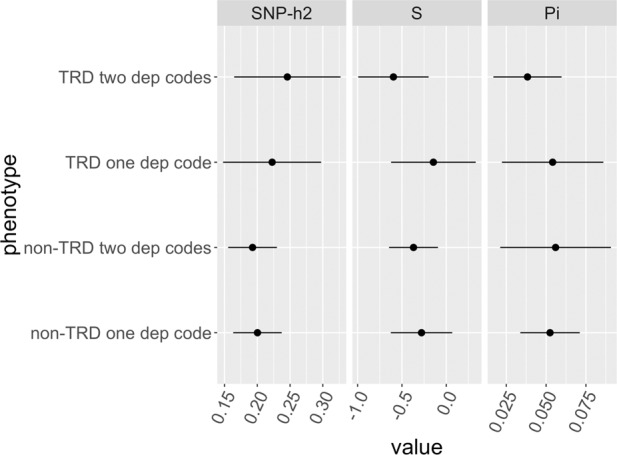
GCTB SNP heritability estimates in UKB. GCTB estimates of SNP-based heritability (SNP-h2), negative selection (S), and polygenicity (proportion of variants with non-zero effects, Pi), for the stringent classification of TRD and non-TRD (≥two diagnostic codes for depression), and a less stringent classification including cases with at least one diagnostic code for depression in UK Biobank.

We estimated the *h*^*2*^_*SNP*_ of TRD vs healthy controls and non-TRD vs healthy controls reducing the stringency by additionally including those with one diagnostic code for depression (2693 with TRD and 23,004 without TRD after quality control). GCTB *h*^*2*^_*SNP*_ of TRD and non-TRD remained similar (Fig. 5). However, the *S* parameter (an estimate of negative natural selection) suggested a different genetic architecture: using at least two diagnostic codes to define MDD, both TRD and non-TRD showed *S* values significantly different from zero (*p* = 0.003 and 0.009, respectively), but not using at least one diagnostic code (Fig. 5).

Without screening controls for disorders other than MDD, the *h*^*2*^_*SNP*_ of non-TRD vs healthy controls was 0.174 (SE = 0.018) and the *h*^*2*^_*SNP*_ of TRD vs healthy controls was 0.233 (SE = 0.040); *S* was not significantly different from zero for both phenotypes. These *h*^*2*^_*SNP*_ would be 0.136 (0.014) and 0.198 (0.034), respectively, if we consider the prevalence of having at least one code for depression instead of two as corresponding to the population prevalence of MDD from the literature.

### Genetic correlations with other traits and PRS results in UKB

A total of 2165 TRD and 14,207 non-TRD participants were included after quality control (Supplementary Methods). There were no genome-wide significant loci associated with TRD vs non-TRD (Supplementary Table 10). LDSC intercept was ~1 for all comparisons, suggesting no confounding factors. TRD vs healthy controls and non-TRD vs healthy controls had similar *r*_*g*_ with other traits; in both cases, *r*_*g*_ were particularly strong and positive with MDD, depressive symptoms and neuroticism (Supplementary Fig. 8; Supplementary Table 11). TRD vs non-TRD did not show significant *r*_*g*_ with other traits, but it had nominally positive (*p* < 0.05) *r*_*g*_ with MDD, depressive symptoms, schizophrenia, bipolar disorder, attention-deficit hyperactivity disorder (ADHD) and insomnia, and negative *r*_*g*_ with subjective wellbeing, childhood IQ and intelligence.

Based on the *r*_*g*_ of TRD vs non TRD with other traits and a previous study [6], we calculated the PRS of eight traits to be tested for association with this phenotype: MDD [16], schizophrenia [34], bipolar disorder [35], ADHD [36], subjective wellbeing [37], childhood IQ [38], intelligence [39] and neuroticism [40]. For subjective wellbeing and intelligence GWAS, there was an overlap with UKB, therefore we excluded the overlapping individuals from the target sample to avoid potential inflation of results (we conservatively excluded those with non-missing data for the phenotypes used in these GWAS, leaving a sample of 1637 and 1310 individuals with TRD, and 10244 and 8227 with non-TRD, for wellbeing and intelligence, respectively). We could not consider the available GWAS of insomnia [41, 42], as the overlap with our target sample was almost complete (16345 participants). All PRS tested had adequate power of over 80% (Supplementary Table 12). The PRS of ADHD was significantly associated with TRD vs non-TRD (OR = 1.09 [1.04–1.14]), with consistent results across different *P*_*T*_ (Supplementary Fig. 9), while the other PRS did not show an effect after Bonferroni correction (Supplementary Fig. 10; Supplementary Table 13A). We tested if the association between ADHD PRS and TRD was modified by adding as covariates each PRS with a nominal effect on TRD (*p* < 0.05, neuroticism, subjective wellbeing and intelligence). We found that this association with ADHD PRS had similar effect sizes when adjusting for neuroticism and subjective wellbeing PRS, while it was notably absent when adding intelligence PRS to the model (Supplementary Table 13B).

## Discussion

### Main findings

This study provides the first large scale population assessment of the genetic and clinical-demographic characteristics of TRD. Based on our phenotype cross-validation in UKB, we demonstrated that MDD can be reliably defined using primary care records and these data can be included in future studies of MDD. The use of least two diagnostic codes for depression to define MDD appears a conservative choice since it increased the phenotypic overlap with the most of the other depression measures in UKB compared to using at least one diagnostic code, though the association with MDD PRS was similar. Together with digital phenotyping, EHR currently represent the most viable option to perform pharmacogenetics studies of antidepressant response with adequate power; the clinical characteristics of the TRD group vs non-TRD were in line with the previous literature (see below) and consistent between the two analysed cohorts, confirming the validity of our TRD phenotype.

The prevalence of MDD found in EXCEED and UKB was 14.2% and 8.7%, respectively, which was similar to the previously reported lifetime prevalence of MDD (10.8%) [28]. The increasing number of antidepressant prescriptions across time reflects the increasing completeness of primary care EHR [18], but also captures real trends. For example, antidepressant prescriptions increased by 10.2% from 2003 to 2004, reflecting a general increase across time, but this flattened to an increase of only 2.6% in 2005, as a probable consequence of the “black box” warning on the risk of antidepressant-induced suicidality in 2004 [43]. The different course of depression diagnoses per year compared to antidepressants prescriptions reflects the fact that diagnostic codes were often not repeated multiple times across years.

Among individuals with MDD, those with TRD showed clinical features suggestive of a more severe disease. Compared to those with non-TRD, they had higher frequencies of longstanding illnesses and psychiatric comorbidities, together with more frequent use of primary care services as shown by clinical and prescription records, and lower SES, in line with previous studies [44, 45]. The association between low frequency or absence of alcohol drinking in TRD vs non-TRD could have been explained by the reported difference in SES, but it persisted after adjusting for education and income, suggesting that SES does not fully account for this finding (Supplementary Table 5C). Higher risk of chronic medical diseases as well as overweight and obesity have been previously associated with TRD [3, 44, 46], but in our results the increased risk of cardio-metabolic comorbidities in TRD was no longer significant after adjusting for BMI. Individuals with TRD also reported less frequent moderate physical activity (Supplementary Table 5C), which may contribute to their insufficient response to antidepressants as well as to their medical comorbidities [47].

In terms of personality traits, participants with TRD compared with non-TRD showed higher neuroticism, as previously described [48], and perceived loneliness, but did not have higher probability of living alone or receiving less visits from family/friends. Higher frequency of irritability and mood swings in TRD vs non-TRD (Supplementary Table 5B) supports the hypothesis of a predisposition towards bipolar disorder in TRD [48]. This hypothesis was consistent with a positive *r*_*g*_ of TRD vs non-TRD with bipolar disorder, but there were no differences in the PRS or in frequency of reported risk-taking behaviours. All the tested psychiatric comorbidities were more common in TRD than non-TRD, particularly anxiety disorders, as well as psychotropic drug polypharmacotherapy, in line with previous studies [3, 44, 48, 49].

Our results suggested that TRD vs healthy controls has a similar *h*^*2*^_*SNP*_ compared with non-TRD vs healthy controls; both these phenotypes may have a different genetic architecture when defined in subjects having at least one diagnostic code for depression rather than at least two, but non-significantly different *h*^*2*^_*SNP*_. In participants having at least two diagnostic codes for depression compared to those having at least one we found indeed a negative GCTB *S* parameter, that was proposed as a marker of negative natural selection, and a broad definition of depression did not show a negative *S* according to a previous study [30]. *S* was not significantly different from zero when considering controls screened for MDD but not for other psychiatric disorders, while *h*^*2*^_*SNP*_ was similar.

Our *h*^*2*^_*SN*P_ of TRD vs healthy controls was similar to results from the largest 23AndMe GWAS of TRD [12]. For TRD vs non TRD, results from both studies were also similar, and our study had sufficient power to show that the *h*^*2*^_*SN*P_ of TRD vs non-TRD was significantly different from zero (0.077 [SE = 0.02] in this study; 0.0779 [SE = 0.04] for [12]), suggesting that TRD may have higher *h*^*2*^_*SN*P_ than non-TRD. The *r*_*g*_ of TRD with non-TRD was significantly different from one, additionally suggesting that TRD and non-TRD have only partially overlapping genetics. However, no significant difference was evident when comparing the *h*^*2*^_*SN*P_ of each group vs healthy controls, in line with the 23AndMe GWAS [12], as the SE of TRD vs controls *h*^*2*^_*SN*P_ was relatively large.

*R*_*g*_ with other traits and PRS analyses supported a stronger shared genetic predisposition between TRD and ADHD, compared to non-TRD. In MDD cases, undetected ADHD was shown to be associated with lack of response to selective serotonin reuptake inhibitors—and a higher number of medications [50]. Interestingly, 17–22% of adults attending psychiatric outpatient clinics for conditions other than ADHD were found to suffer from this disorder; however, fewer than 20% of adults with ADHD are diagnosed and/or treated by psychiatrists [51]. Therefore, undiagnosed ADHD or a past diagnosis of the disorder should be assessed in patients with TRD. In UKB, ADHD diagnosis could not be reliably assessed in primary care data (prevalence < 0.01%), probably due to the lack of registration of childhood diagnoses and low awareness of the manifestations of ADHD in adults [51].

### Limitations

EHR used to define TRD and non-TRD groups do not necessarily reflect complete information regarding antidepressant prescription, particularly for prescriptions issued before 1990s. We had no direct measure of treatment adherence; however, our TRD prevalence was in the range of previous estimates and we considered the time between all consecutive prescriptions to assess this issue. The lack of a standardised diagnostic assessment together with the observed variety of diagnoses (partly based on old nosology) may have led to the inclusion of cases with depressive disorders other than MDD; however, the prevalence in line with previous studies, the overlap with other measures of unipolar depression and the genetic overlap with MDD suggest that this issue was mild at most. We did not have a direct measure of treatment response, but we assumed that a switch to a different antidepressant after at least 6 weeks of treatment was indicative of lack of efficacy, since a switch due to side effects would probably happen earlier [6] and switching is the most common strategy for TRD management [52]. Prescribed daily medication dose was not available, therefore was not used for the definition of TRD, and subtherapeutic doses may have inflated the observed TRD rate. Primary care data were available only in about half of UKB participants, therefore there was limited overlap with variables assessed in other subsets of the sample. The low prevalence of some disorders in primary care data, such as personality disorders, is likely caused by the lack of training of general practitioners (GP) for assessing these diagnoses, as shown by the low agreement between GP diagnosis and structured interviews, and the previously reported low prevalence in primary care [53].

Regarding the genetic part of the study, we had inadequate power to identify variants associated with TRD versus non-TRD at the genome-wide level [54]. Though there was a nominally higher *r*_*g*_ with insomnia for TRD vs. non-TRD, we did not test the PRS of insomnia because the two main GWAS available had a very high overlap with our target sample [41, 42].

## Conclusions

This study has demonstrated that MDD and TRD can be reliably defined according to EHR of primary care events, therefore EHR represent an exciting opportunity for large genetic and pharmacogenetics studies: we provided a framework that can be applied to other cohorts with similar data and lead to highly powered meta-analyses.

Our results suggested that TRD has partially distinct genetic and clinical-demographic characteristics compared with non-TRD that may be helpful to identify patients who should be considered for referral to secondary care. Social policies should promote awareness of the factors associated with TRD and its negative consequences on health, as well as aim to reduce inequalities related to SES as these are likely to impact on the risk of TRD.

## Supplementary information

Supplementary Materials
