# Supplementary material for: Genetic and clinical characteristics of treatment-resistant depression using primary care records in two UK cohorts
Source: Mol Psychiatry. 2021 Mar 22;26(7):3363–73. doi: 10.1038/s41380-021-01062-9 (PMC8505242; doi:10.1038/s41380-021-01062-9)

Genetic and clinical characteristics of treatment-resistant depression using primary care records in two UK cohorts

Supplementary Materials

Table of contents

Supplementary Methods……… p. 1
Supplementary Table 1……… p. 8
Supplementary Table 2……… p. 9
Supplementary Table 3……… p. 11
Supplementary Table 4……… p. 13
Supplementary Table 5……… p. 14
Supplementary Table 6……… p. 21
Supplementary Table 7……… p. 22
Supplementary Table 8……… p. 23
Supplementary Table 9……… p. 26
Supplementary Table 10……… p. 28
Supplementary Table 11……… p. 31
Supplementary Table 12……… p. 33
Supplementary Table 13……… p. 35
Supplementary Figure 1……… p. 36
Supplementary Figure 2……… p. 38
Supplementary Figure 3……… p. 39
Supplementary Figure 4……… p. 40
Supplementary Figure 5……… p. 41
Supplementary Figure 6……… p. 43
Supplementary Figure 7……… p. 44
Supplementary Figure 8……… p. 50
Supplementary Figure 9……… p. 51
Supplementary Figure 10……… p. 52

Supplementary Methods

1. Primary care data in UK Biobank (UKB)

There is currently no national system for collecting or sharing primary care data in the UK. UKB has been liaising with various data suppliers and other intermediaries (including the main primary care computer system suppliers in England) to obtain primary care data for UKB participants, all of whom have provided written consent for linkage to their health-related records. To date, coded data have been obtained for approximately 45% of the UK Biobank cohort (~230,000 participants) and are now available as part of this interim release. UKB is currently in the process of securing access to data

for the remaining cohort, mainly for participants registered with EMIS (a computer system supplier to the NHS) practices across England (1).
The dataset contains variables that are considered the most important for epidemiological research: coded clinical events (including diagnoses, history, symptoms, lab results, procedures), prescriptions (i.e. medications that are prescribed but not necessarily dispensed) and a range of administrative codes (e.g. referrals to specialist hospital clinics). Non-coded, unstructured data (e.g. free-text entries, referral letters) are not included (1).
The primary care computer system suppliers have adopted different coding classifications as part of their underlying data schema. In addition to these coding variations for clinical events, the different system suppliers use a range of coding classifications for prescriptions, as reported in the following table:

**Country**	**GP Computer System Supplier**	**Approx no. of UKB participants**	**Clinical coding classification**	**Prescription coding classification**
Scotland	EMIS / Vision	27,000	Read v2	- Read v2<br>- British National Formulary (BNF)
Wales	EMIS / Vision	21,000	Read v2	Read v2
England	TTP	165,000	Clinical Terms Version 3 (CTV3 or Read v3)	BNF
	Vision	18,000	Read v2	- Read v2<br>- Dictionary of Medicines and Devices (dm+d)

Read codes are a coded thesaurus of clinical terms used in primary care since 1985. There are two versions: version 2 (Read v2) and version 3 (CTV3 or Read v3). Both provide a standard vocabulary for clinicians to record patient findings and procedures. Read v2 and CTV3, together with a UK Read code browser, are available via the NHS Digital Technology Reference Data Update Distribution (TRUD) website4. Read v2 and CTV3 were last updated in April 2016 and April 2018, respectively. Both versions are now deprecated and no further updates will occur. From April 2018, SNOMED CT was introduced into primary care in a phased approach and it is intended by April 2020 that SNOMED CT will be fully incorporated across the wider NHS, including codes related to prescriptions (1).
The BNF is the standard list of medicines, dressings and appliances prescribed in the UK. It is published as a reference guide in both online and paper versions and contains information on, for example, dose, side effects and price for over 70,000 items. Code lists are updated annually and can be downloaded from the NHS Business Services Authority (NHSBSA) (1).
The prescription data from Vision (England) contains dm+d codes (as well as Read v2 codes) to record medicines prescribed to patients. The dm+d dictionary has been developed for use throughout the NHS (primary and secondary care) to identify specific medicines and devices used

in the treatment of patients and consists of a dictionary containing unique identifiers and associated text descriptions (1).
In order to facilitate research on these data, clinical code lists have been compiled from TRUD and NHSBSA (Appendix C) [see Resource 592 on UK Biobank data showcase]. TRUD has historically provided information on how to map from Read v2 and CTV3 to other clinical coding systems (1).
Information on participant registrations varies by data supplier, in that Vision (England) provided a single registration record per person while the other suppliers provided multiple records per participant, and a small number of participants with data in the TPP extract do not have a registration record. Therefore, variable numbers of registration records are included in this release, reflecting the providers' extracts. The start date of coverage is not known for all participants, nor is the completeness of coverage of their primary care health records until the extract date (1).
Data were extracted from supplier's computer systems using different approaches, in each case making a single extract date or cut-off point impossible to determine. For more information see (1).

We classified Read v2 and CTV3 clinical codes in diagnostic groups (e.g. depressive disorders, bipolar disorders, anxiety disorders) and we linked Read v2 clinical codes to the corresponding CTV3 clinical codes. Prescription codes were reported not only according to different classifications but within the same classification system different formats were used (e.g. BNF codes sometimes included dots while other times they didn't), therefore we manually inspected samples of the data to find irregularities and extract the records of interest accordingly. For some prescription records the only information provided was medication code and issue date, though in most cases also the drug name was included as reported in the drug label (brand or generic). Therefore, we had to annotate prescription records with medication chemical name and class (e.g. antidepressant, antipsychotic) using as reference information provided by NHS websites (dm+d browser; British National Formulary). In order to facilitate the extraction of data by other investigators, these annotated tables are available at: https://doi.org/10.1101/2020.08.24.20178715.
Where clinical event or prescription date preceded or matched participant date of birth, it was in the year of their birth, or it was in the future, it has been altered to some predefined values in UKB data (01/01/1901, 02/02/1902, 03/03/1903 and 07/07/2037), and these values were set to missing for the analyses of this study.

2. Other measures of depression in UKB

Five other measures of depression were considered in UKB for comparison with primary care-defined depression (Figure 1):

1) Lifetime depression defined based on the Composite International Diagnostic Interview Short Form (CIDI-SF) (2) that was part of the Mental Health Questionnaire (MHQ). Criteria for lifetime major depressive episode were in accordance with DSM-V. The full CIDI is a validated measure of depression, demonstrated to have good concordance with direct clinical assessment (3).
2) Lifetime depression based on hospital diagnosis (ICD-10 codes F32-F33-F34.1), considering both main ICD-10 diagnoses (data field 41202) and secondary ICD-10 diagnoses (data field

41204). Individuals having at least one ICD-10 code but no ICD-10 code for a depressive disorder were considered as having no lifetime depression based on this measure.

3) Self-reported depression diagnosed by a professional (data field 20544). This corresponded to the question: “Have you been diagnosed with one or more of the following mental health problems by a professional, even if you don't have it currently?”.
4) Help-seeking definition of depression according to the questions “Have you ever seen a general practitioner (GP) for nerves, anxiety, tension or depression?” (data field 2090) and “Have you ever seen a psychiatrist for nerves, anxiety, tension or depression?” (data field 2100). Individuals who answered ‘yes’ to at least one of these two questions were considered as having a current or lifetime depression.
5) Smith et al. definition of depression (4). This was defined as probable lifetime depression based on a combination of measures (items relating to the lifetime experience of minor and major depression, items from the Patient Health Questionnaire (PHQ) and items on help-seeking for mental health). In detail, this phenotype was defined as having satisfied at least one of the following two groups of criteria: a) ever felt depressed/down for a whole week; plus at least two weeks duration of depression; plus ever seen a GP or psychiatrist for “nerves, anxiety, tension or depression”; OR b) ever anhedonic for a whole week; plus at least two weeks duration of depression; plus ever seen a GP or psychiatrist for “nerves, anxiety, tension or depression”.

3. Genotyping, quality control and imputation

3.1. UK biobank

Genome-wide genotyping on all UK Biobank participants was performed using two highly overlapping arrays covering ~800,000 markers. Autosomal genotype data underwent centralised quality control to adjust for possible array effects, batch effects, plate effects, and departures from Hardy-Weinberg equilibrium (HWE) (5). SNPs were further excluded based on missingness (> 0.02) and on Hardy Weinberg equilibrium (p < 10-8). Individuals were removed for high levels of missingness (> 0.05) or abnormal heterozygosity (as defined during centralised quality control), relatedness of up to third-degree kinship (KING r < 0.044 (6)) or phenotypic and genotypic gender discordance. Population structure within the UK Biobank cohort was assessed using principal component analysis, with European ancestry defined by 4-means clustering on the first two genetic principal components (7).

A two-stage imputation was performed using the Haplotype Reference Consortium (HRC) and UK10K reference panels (5) (8) (9). Poor imputed variants were excluded (INFO ≤ 0.4) (8).

3.2. EXCEED cohort

Over 60% of EXCEED participants have been genotyped using the Affymetrix UK Biobank Axiom Array. Data were available in 5216 participants after quality control, with imputation to the Haplotype Reference Consortium (HRC) panel (8). Variants for the polygenic risk score analysis were limited to common variants (minor allele frequency >0.01) that were directly genotyped. Variants were further excluded based on missingness (>0.05) and on Hardy-Weinberg equilibrium $P < 1\times10^{-6}$.

Variants were checked for plate effects, i.e. variants that have significantly different minor allele counts on a particular plate compared to all other plates. A χ^2 test was used to compare the minor allele count of a variant on each plate to its minor allele count on all other plates. A *P*-value of 1×10^{-12} was used to indicate a significant plate effect, and variants were excluded or set to missing according to how many plates a variant showed a significant plate effect.
Individuals were excluded for high levels of missingness (>0.05) or abnormal ancestry-adjusted heterozygosity rate (more than 6 standard deviations from the mean), as conducted by UK Biobank (5), relatedness of up to third-degree kinship (according to IBD analysis in PLINK 1.90 (10) (PI_HAT >0.125), the individual with the highest missingness was excluded from each related pairing), or phenotypic and genotypic gender discordance. Population structure was assessed using principal components analysis. The starting cluster centres for *k*-means clustering were defined as the mean principal components for each of the 5 1000 Genomes Phase 3 super populations (EUR, AFR, EAS, SAS, AMR). This was done as unsupervised *k*-means clustering (cluster centres randomly selected) was unsuccessful for the EXCEED samples. European ancestry was, thus, defined by 5-means clustering of the first 4 principal components.

4. Polygenic risk scores

In both cohorts, polygenic risk scores (PRS) were calculated using PRSice v.2 (11) and genotyped variants. PRSice computes scores in an independent (target) sample by calculating the weighted sum of trait-associated alleles using summary data from GWAS discovery samples. SNPs in linkage disequilibrium (r2 ≥ 0.1 [250-kb window]) were removed. We used the default average option that calculates the ratio between the PRS and the number of alleles included in each individual; PRS were standardised (mean=0, SD=1). PRS were calculated at 11 p-value thresholds P_T (5e-8, 1e-5, 1e-3, 0.01, 0.05, 0.1, 0.2, 0.3, 0.4, 0.5, 1) and the most predictive PT was selected. Logistic regression models were used to estimate associations between the phenotype and each PRS adjusting for covariates of six genetic ancestry principal components, assessment centre and batch effects in UKB, and six genetic ancestry principal components and primary care practice in EXCEED. The proportion of variance explained by PRS on the liability scale was estimated according to Lee et al. (12), assuming MDD prevalence of 10.8% for case-control comparisons (13), and using the observed scale for case only comparisons. For case-control comparisons using different definitions of major depressive disorder (MDD) in UKB, for converting Nagelkerke R^2 to the liability scale we considered the relative frequency of each MDD phenotype compared to our primary care-defined MDD (at least two diagnostic codes for a depressive disorder), and we multiplied it by the prevalence reported in the general population (10.8%).
For determining the adequacy of the base GWAS summary statistics to generate PRS for prediction of TRD vs non-TRD, we performed a power analyses using the R package "avengeme". The covariance between genetic effect sizes in the base and target samples (cov) was calculated as $cov = r_g \times \sqrt{(h^2{}_{SNP}base \times h^2{}_{SNP}target)}$, where r_g is the genetic correlation between the trait considered in the base sample and the target sample (TRD vs non-TRD), $h^2{}_{SNP}base$ is the SNP-based heritability of the trait considered in the base sample and $h^2{}_{SNP}target$ is the SNP-based heritability of TRD vs non-TRD. The considered alpha value was 5.88e-4 (Bonferroni correction considering the number of traits and of P_T). The proportion of variants with no effect on the training trait was set to

0.95. For subjective wellbeing (14) and intelligence (15), the power was calculated after the exclusion from the target sample of individuals overlapping with the base sample (leaving a sample of 1637 and 1310 individuals with TRD, and 10244 and 8227 with non-TRD, for wellbeing and intelligence, respectively). We identified these individuals in a conservative way, as those who had non-missing values for the items used to define the phenotypes of the respective GWAS (field 20458: general happiness; field 20016: fluid intelligence/reasoning). The results of the power analysis are reported in Supplementary Table 12.

5. Heritability estimates in UK Biobank

For the estimation of heritability (h^2_{SNP}) of TRD and non-TRD in UKB, we used genome-wide complex trait analysis software v.1.93.1beta (GCTA) (16). The genetic relationship matrix was adjusted for incomplete tagging of causal SNPs and we further excluded related individuals using a grm-cut off of 0.05. We included 11,188 healthy controls (no psychiatric diagnoses) as they provided adequate power assuming a prevalence of 0.02 of TRD in the population and heritability of 0.10 for MDD (17),(18). We also calculated h^2_{SNP} using Genome-wide Complex Trait Bayesian (GCTB) Bayes S method. GCTB uses the data to estimate polygenicity and calculates the relationship between effect size and MAF (*S*) which can be used to detect signatures of natural selection (19). For all analyses, six genetic ancestry principal components, assessment centre and batch effects were included as covariates.

References

1. UK Biobank. Primary Care Linked Data [Internet]. 2019. Available from: http://biobank.ndph.ox.ac.uk/showcase/showcase/docs/primary_care_data.pdf
2. Kessler RC, Andrews G, Mroczek D, Ustun B, Wittchen H-U. The World Health Organization Composite International Diagnostic Interview short-form (CIDI-SF). Int J Methods Psychiatr Res. 1998 Nov;7(4):171–85.
3. Haro JM, Arbabzadeh-Bouchez S, Brugha TS, De Girolamo G, Guyer ME, Jin R, et al. Concordance of the Composite International Diagnostic Interview Version 3.0 (CIDI 3.0) with standardized clinical assessments in the WHO World Mental Health Surveys. Int J Methods Psychiatr Res. 2006 Dec;15(4):167–80.
4. Smith DJ, Nicholl BI, Cullen B, Martin D, Ul-Haq Z, Evans J, et al. Prevalence and characteristics of probable major depression and bipolar disorder within UK biobank: cross-sectional study of 172,751 participants. PloS One. 2013;8(11):e75362.
5. Bycroft C, Freeman C, Petkova D, Band G, Elliott LT, Sharp K, et al. The UK Biobank resource with deep phenotyping and genomic data. Nature. 2018 Oct;562(7726):203–9.
6. Manichaikul A, Mychaleckyj JC, Rich SS, Daly K, Sale M, Chen W-M. Robust relationship inference in genome-wide association studies. Bioinforma Oxf Engl. 2010 Nov 15;26(22):2867–73.
7. Warren HR, Evangelou E, Cabrera CP, Gao H, Ren M, Mifsud B, et al. Genome-wide association analysis identifies novel blood pressure loci and offers biological insights into cardiovascular risk. Nat Genet. 2017 Mar;49(3):403–15.
8. McCarthy S, Das S, Kretzschmar W, Delaneau O, Wood AR, Teumer A, et al. A reference panel of 64,976 haplotypes for genotype imputation. Nat Genet. 2016 Oct;48(10):1279–83.
9. UK10K Consortium, Walter K, Min JL, Huang J, Crooks L, Memari Y, et al. The UK10K project identifies rare variants in health and disease. Nature. 2015 Oct 1;526(7571):82–90.
10. Chang CC, Chow CC, Tellier LC, Vattikuti S, Purcell SM, Lee JJ. Second-generation PLINK: rising to the challenge of larger and richer datasets. GigaScience. 2015;4:7.
11. Choi SW, O'Reilly PF. PRSice-2: Polygenic Risk Score software for biobank-scale data. GigaScience.

2019 Jul 1;8(7).
12. Lee SH, Goddard ME, Wray NR, Visscher PM. A Better Coefficient of Determination for Genetic Profile Analysis: A Better Coefficient of Determination. Genet Epidemiol. 2012 Apr;36(3):214–24.
13. Lim GY, Tam WW, Lu Y, Ho CS, Zhang MW, Ho RC. Prevalence of Depression in the Community from 30 Countries between 1994 and 2014. Sci Rep. 2018 12;8(1):2861.
14. Okbay A, Baselmans BML, De Neve J-E, Turley P, Nivard MG, Fontana MA, et al. Genetic variants associated with subjective well-being, depressive symptoms, and neuroticism identified through genome-wide analyses. Nat Genet. 2016;48(6):624–33.
15. Savage JE, Jansen PR, Stringer S, Watanabe K, Bryois J, de Leeuw CA, et al. Genome-wide association meta-analysis in 269,867 individuals identifies new genetic and functional links to intelligence. Nat Genet. 2018;50(7):912–9.
16. Yang J, Lee SH, Goddard ME, Visscher PM. GCTA: a tool for genome-wide complex trait analysis. Am J Hum Genet. 2011 Jan 7;88(1):76–82.
17. Visscher PM, Hemani G, Vinkhuyzen AAE, Chen G-B, Lee SH, Wray NR, et al. Statistical power to detect genetic (co)variance of complex traits using SNP data in unrelated samples. PLoS Genet. 2014 Apr;10(4):e1004269.
18. Wray NR, Ripke S, Mattheisen M, Trzaskowski M, Byrne EM, Abdellaoui A, et al. Genome-wide association analyses identify 44 risk variants and refine the genetic architecture of major depression. Nat Genet. 2018 May;50(5):668–81.
19. Zeng J, de Vlaming R, Wu Y, Robinson MR, Lloyd-Jones LR, Yengo L, et al. Signatures of negative selection in the genetic architecture of human complex traits. Nat Genet. 2018;50(5):746–53.

Supplementary Table 1: genome-wide summary statistics used to create the polygenic risk scores (PRSs) in UKB and EXCEED (in EXCEED only PRS for major depressive disorder, schizophrenia and bipolar disorder were tested for association with depression defined using primary care data).

Trait	Type of phenotype	N cases	N controls	Ethnicity	Reference (PubMed ID)
**Psychiatric disorders**					
Major depressive disorder*	Binary	45,591	97,674	European	29700475
Schizophrenia	Binary	40,675	64,643	European	29483656
Bipolar disorder	Binary	20,352	31,358	European	31043756
Attention-deficit hyperactivity disorder	Binary	20,183	35,191	European	30478444
**Personality and related traits**					
Neuroticism	Continuous	63,030		European	25993607
Subjective wellbeing**	Continuous	298,420		European	27089181
**Cognitive traits**					
Intelligence**	Continuous	269,867		European	29942086
Childhood IQ	Continuous	12,441		European	23358156

* Subjects included in UK Biobank and 23AndMe were excluded from the analyses

** The individuals overlapping with UK Biobank were excluded from the target sample, see paragraph 4. of the Supplementary Methods

Supplementary Table 2: genome-wide summary statistics used to test genetic correlations with treatment-resistant depression (TRD) and non-TRD using linkage disequilibrium score regression (LDSC) in UKB. ADHD=attention deficit hyperactivity disorder; ASD=autism spectrum disorders.

Trait	Type of phenotype	N cases	N controls	Ethnicity	Reference (PubMed ID)
**Psychiatric disorders**					
Major depressive disorder*^	Binary	45,591	97,674	European	29700475
Depressive symptoms	Continuous	105,739		European	27089181
	Binary	16,471	58,656		
Bipolar disorder^	Binary	20,352	31,358	European	31043756
Schizophrenia	Binary	36,989	113,075	European	25056061
ADHD	Continuous	17,666		European	27663945
ASD	Binary	18,381	27,969	European	30804558
Anorexia nervosa^	Binary	16,992	55,525	European	31308545
PGC cross-disorder study	Binary	33,332	27,888	European	23453885
**Personality and related traits**					
Neuroticism	Continuous	170,911		European	27089181
Openness to experience	Continuous	17,375		European	21173776
Consciousness	Continuous	17,375		European	21173776
Subjective wellbeing	Continuous	298,420		European	27089181
**Sleep-related traits**					
Insomnia	Binary	32,155	26,973	European	27992416
Sleep duration	Continuous	128,266		European	27494321
Chronotype (morningness)	Continuous	128,266		European	27494321
**Cognitive and related traits**					
Childhood IQ	Continuous	17,989		European	23358156
Intelligence^	Continuous	269,867		European	29942086
Years of schooling	Continuous	293,723		European	27225129
College completion	Continuous	126,559		European	23722424
**Cardio-metabolic and related traits**					
LDL cholesterol	Continuous	95,454		European	20686565
HDL cholesterol	Continuous	99,900		European	20686565
Triglycerides	Continuous	96,598		European	20686565
Type 2 diabetes	Binary	34,840	114,981	European	22885922
Coronary artery disease	Binary	60,801	123,504	European and south Asian	26343387
Ever smoked	Binary	41,951	32,102	European	20418890
Obesity class 1	Binary	55,229	104,894	European	23563607
Obesity class 2	Binary	15,334	97,858	European	23563607
Obesity class 3	Binary	3,986	67,010	European	23563607
BMI	Continuous	249,796		European	20935630
Waist-to-hip ratio	Continuous	224,459		European	25673412

* Subjects from UK Biobank and 23AndMe were excluded

^ The genetic correlations with the TRD phenotypes were not estimated using LD Hub for these traits but using the software locally because of the availability of significantly smaller datasets on LD Hub compared to the most recent studies

Definitions of obesity:
Obesity class 1: BMI ≥30 kg/m2
Obesity class 2: BMI ≥35 kg/m2
Obesity class 3: BMI ≥40 kg/m2

Supplementary Table 3: **(A)** results of the association analyses between the PRS of major depressive disorder (MDD), bipolar disorder and schizophrenia with depression diagnosis in UK Biobank primary care data (at least two or more diagnostic codes or at least one diagnostic code). For each measure of MDD, the number of cases and controls included in the analysis is shown in parenthesis. Results referred to the best p threshold (P_T) are shown, but they were consistent across the different P_T tested. OR are referred to one standard deviation increase in the PRS. Nagelkerke R2 was reported on the liability scale considering a prevalence of 10.8% (13) for the MDD definition based on primary care records (≥ two GP codes depression), while for the other MDD phenotypes we considered the relative prevalence compared to ≥ two GP codes depression (e.g. ≥ one GP depression code had a prevalence (16.03%) that was 1.85 times the prevalence of ≥ two GP depression codes (8.68%): 10.8% x 1.85 = 19.9%). After Bonferroni correction, alpha=2.16e-4. **(B)** Results of the association analyses between the PRS of MDD, bipolar disorder and schizophrenia with depression diagnosis in the EXCEED sample. After Bonferroni correction, alpha=1.52e-3.

A

**Base data**	**Target trait**	**Best P_T**	**E (SE)**	**P**	**OR (95% CI)**	**Nagelkerke R2**
Major depressive disorder	≥ two GP codes depression (17,807 vs 130,252)	0.3	0.15 (0.008)	1.89e-71	1.16 (1.14-1.17)	0.0055
Bipolar disorder		0.5	0.07 (0.008)	2.78e-17	1.07 (1.06-1.09)	0.0012
Schizophrenia		1	0.09 (0.008)	3.96e-25	1.09 (1.07-1.11)	0.0018
Major depressive disorder	≥ one GP code depression (30,822 vs. 130,252)	0.5	0.14 (0.006)	2.21e-100	1.15 (1.13-1.16)	0.0059
Bipolar disorder		0.05	0.06 (0.006)	5.04e-22	1.06 (1.05-1.08)	0.0012
Schizophrenia		0.3	0.08 (0.007)	4.51e-34	1.08 (1.07-1.10)	0.0019
Major depressive disorder	CIDI-SF depression (25,842 vs 79,981)	0.4	0.13 (0.007)	6.82e-75	1.14 (1.13-1.16)	0.0060
Bipolar disorder		0.3	0.08 (0.007)	1.82e-26	1.08 (1.07-1.10)	0.0020
Schizophrenia		0.5	0.10 (0.007)	4.26e-43	1.11 (1.09-1.13)	0.0034
Major depressive disorder	ICD depression (13,129 vs 290,380)	0.2	0.15 (0.009)	4.55e-64	1.16 (1.14-1.19)	0.0049
Bipolar disorder		0.3	0.09 (0.009)	4.11e-20	1.09 (1.07-1.11)	0.0014
Schizophrenia		1	0.08 (0.009)	2.25e-18	1.08 (1.07-1.10)	0.0013
Major depressive disorder	Smith depression (23,760 vs 63,610)	0.5	0.14 (0.008)	1.68e-70	1.15 (1.13-1.16)	0.0067
Bipolar disorder		0.1	0.08 (0.008)	1.81e-26	1.09 (1.07-1.10)	0.0024
Schizophrenia		0.4	0.10 (0.008)	1.38e-39	1.11 (1.09-1.13)	0.0037
Major depressive disorder	Self-reported depression (23,093 vs 81,662)	0.5	0.15 (0.008)	2.02e-92	1.17 (1.15-1.18)	0.0079
Bipolar disorder		0.5	0.08 (0.008)	1.25e-22	1.08 (1.06-1.10)	0.0018
Schizophrenia		0.4	0.09 (0.008)	6.32e-30	1.09 (1.08-1.11)	0.0024

Major depressive disorder	Seen GP or psychiatrist (130,458 vs 237,166)	0.3	0.13 (0.003)	<5e-324	1.14 (1.13-1.15)	0.0067
Bipolar disorder		0.4	0.08 (0.004)	1.62e-108	1.08 (1.08-1.09)	0.0023
Schizophrenia		0.4	0.10 (0.004)	2.33e-164	1.10 (1.10-1.11)	0.0034

B

**Base data**	**Target trait**	**Best P_T**	**E (SE)**	**P**	**OR (95% CI)**	**Nagelkerke R^2**
Major depressive disorder	≥ 2 GP codes for depression (557 vs 2,181)	0.2	0.23 (0.05)	6.05e-6	1.26 (1.14-1.39)	0.0124
Bipolar disorder		0.05	0.14 (0.05)	0.0060	1.15 (1.04-1.26)	0.0045
Schizophrenia		0.05	0.11 (0.05)	0.0276	1.12 (1.01- 1.23)	0.0029

Supplementary Table 4: number of UK Biobank participants having at least two, at least one or zero depression codes in primary care data who endorsed other measures of depression (ICD depression based on hospital records, Smith depression, self-reported (SR) depression diagnosed by a professional, depression according to the Composite International Diagnostic Interview Short Form (CIDI-SF) and help-seeking depression based on having seen a general practitioner (GP) or psychiatrist for depression-anxiety, see Supplementary Methods and Figure 1). For each pair of depression phenotypes (non-primary care vs. primary care), the no. of subjects having depression according to both / no. of subjects who endorsed the primary care measure and had non-missing values for both measures is reported (see also Figure 3). A Fisher's exact test was used to compare the difference in overlap between those having at least two diagnostic codes for depression, one diagnostic code for depression and no diagnostic code for depression (right-hand part of the table). * p values < Bonferroni corrected p value of 5e-3.

Depression measure	Overlap with ≥ two depression diagnostic codes in primary care	Overlap with ≥ one depression diagnostic code in primary care	Overlap with no depression diagnostic code in primary care	Overlap in those with ≥ two vs. ≥ one depression diagnostic code	Overlap in those with ≥ two vs. no depression diagnostic code
ICD depression	3375/16873 (20.00%)	4852/31098 (15.60%)	2440/149122 (1.64%)	OR=1.28 (1.22-1.35), p=3.79e-24*	OR=12.22 (11.57-12.92), p<5e-324*
Smith depression	3644/4869 (74.84%)	6091/8909 (68.37%)	8905/47341 (18.81%)	OR=1.09 (1.04-1.16), p=1.04 e-3*	OR=3.98 (3.79-4.18), p<5e-324*
Self-reported depression	3623/4146 (87.39%)	6133/7602 (80.68%)	6678/50603 (13.20%)	OR=1.08 (1.02-1.15), p=5.20e-3	OR=6.62 (6.29-6.97), p<5e-324*
CIDI-SF depression	2858/4018 (71.13%)	4856/7432 (65.34%)	9704/51541 (18.83%)	OR=1.09 (1.02-1.16), p=5.71e-3	OR=3.78 (3.58-3.98), p<5e-324*
Seen GP or psychiatrist for depression	16567/18872 (87.79%)	28520/34853 (81.83%)	47308/181069 (26.13%)	OR=1.07 (1.05-1.10), p=1.34e-7*	OR=3.36 (3.28-3.44), p<5e-324*

Supplementary Table 5: socio-demographic (**A**), psychiatric clinical (**B**), body max index (BMI), medical diseases and lifestyles (**C**) variables tested for association with treatment-resistant depression (TRD) compared to non-TRD in EXCEED and UK Biobank (UKB). Mean (standard deviation or SD) and number (%) are reported for describing continuous and categorical variables, respectively, and number of missing values (N missing). Analyses were logistic regression (OR (95% CI) are reported, continuous predictors were standardized for easier interpretation and comparison between samples), Kruskal-Wallis test or Cochran-Armitage trend. P-values <5e-324 were reported as range. *significant p-values after Bonferroni correction: alpha=1.43e-3 in UKB (35 variables were tested) and alpha=0.0028 in EXCEED (18 variables were available).

A

		EXCEED			UKB		
**Variable**	**Group**	**Description**	**Effect size**	**p**	**Description**	**Effect size**	**p**
Age at assessment	TRD	57.11 (7.50)	OR=0.92 (0.78-1.09)	0.33	55.75 (7.85)	OR=1.05 (1.01-1.10)	0.017
	Non-TRD	57.80 (8.21)			55.33 (8.01)		
	N missing	38			0		
Sex (F/M)	TRD	115/44 (72.33% F)	OR=1.08 (0.74-1.57)	0.69	1751/679 (72.06% F)	OR=1.18 (1.07-1.30)	5.68e-4*
	Non-TRD	722/298 (70.78% F)			10940/5011 (68.59% F)		
	N missing	0			0		
Ethnicity (white/other)	TRD	141/11 (92.76% white)	OR=0.90 (0.46-1.75)	0.76	2311/106 (95.61% white)	OR=0.85 (0.69-1.05)	0.13
	Non-TRD	924/65 (93.43% white)			15279/594 (96.25% white)		
	N missing	38			91		
Index of multiple deprivation quintile (%) in EXCEED and townsend deprivation index in UKB	TRD	1st quintile: 48 (32.00%)<br>2nd quintile: 15 (10.00%)<br>3rd quintile: 21 (14.00%)<br>4th quintile: 38 (25.33%)<br>5th quintile: 28 (18.67%)	1st quintile: reference level<br><br>2nd quintile: OR=0.34 (0.18, 0.63)<br><br>3rd quintile: OR=0.44 (0.25, 0.77)<br><br>4th quintile: OR=0.51	0.004 (Cochran-Armitage trend)	-0.77 (3.28)	Kruskal-Wallis chi2=11.49	7.02e-4*
	Non-TRD	1st quintile: 163 (16.94%)<br>2nd quintile: 151 (15.70%)<br>3rd quintile: 161 (16.74%)<br>4th quintile: 254 (26.40%)<br>5th quintile: 233 (24.22%)			-1.05 (3.11)		

			(0.32, 0.81)<br><br>5th quintile: OR=0.41 (0.25, 0.68)				
	N missing	67			26		
Number in household (>1/total)	TRD	NA	NA	NA	577/2404 (24.00%)	OR = 0.98 (0.88 – 1.08)	0.65
	Non-TRD	NA			3726/15805 (23.57%)		
	N missing	-			172		
Leisure - social activities (yes/total)	TRD	NA	NA	NA	1511/2421 (62.41%)	OR = 0.89 (0.81 – 0.97)	0.007
	Non-TRD	NA			10377/15860 (65.43%)		
	N missing	-			100		
Able to confide (where 0 is never and 5 is daily)	TRD	NA	NA	NA	3.14 (1.95)	Kruskal-Wallis chi2 = 23.52	1.25e-6*
	Non-TRD	NA			3.34 (1.92)		
	N missing	-			570		
Frequency of visits from family/friends (0-7)	TRD	NA	NA	NA	2.69 (1.23)	Kruskal-Wallis chi2=13.87	0.031
	Non-TRD	NA			2.69 (1.17)		
	N missing	-			152		
Education^, number per category (% per category)	TRD	NA	NA	NA	College: 535 (22.22%)<br>A-levels: 284 (11.79%)<br>0-levels-GCSEs: 547 (22.72%)<br>CSEs: 171 (7.10%)<br>NVQ-HND-HNC: 148 (6.15%)<br>Other professional title: 126 (5.23%)<br>None of the above: 597 (24.79%)	College: OR = 0.64 (0.56 – 0.73)<br><br>A-levels: OR = 0.84 (0.72 – 0.98)<br><br>0-levels-GCSEs: OR = 0.80 (0.70 – 0.91)<br><br>CSEs: OR = 0.86 (0.71 – 1.05)<br><br>NVQ-HND-HNC: OR = 0.70 (0.58 – 0.85)	1.61e-11*<br><br>0.029<br><br>0.00076*<br><br>0.13<br><br>0.00033*
	Non-TRD	NA			College: 4381 (27.84%)<br>A-levels: 1764 (11.21%)<br>0-levels-GCSEs: 3528 (22.42%)<br>CSEs: 1041 (6.62%)		

					NVQ-HND-HNC: 1116 (7.09%)<br>Other professional title: 840 (5.34%)<br>None of the above: 3064 (19.47%)	Other professional title: OR = 0.77 (0.63 – 0.95)<br><br>None of the above: reference level	0.014<br><br>-
	N missing	-			239		
Average household income £, number per category (% per category)	TRD	NA	NA	NA	<18K: 853 (41.98%)<br>18K-30.9K: 534 (26.28%)<br>31K-51.9K: 420 (20.67%)<br>52K-100K: 206 (10.14%)<br>>100K: 19 (0.94%)	<18K: Reference level<br><br>18K-30.9K: OR = 0.72 (0.64 – 0.81)<br><br>31K-51.9K: OR = 0.63 (0.56 – 0.72)<br><br>52K-100K: OR = 0.49 (0.42 – 0.58)<br><br>>100K: OR = 0.26 (0.16 – 0.41)	-<br><br>4.62e-8*<br><br>7.08e-13*<br><br>5.81e-18*<br><br>1.13e-8*
	Non-TRD	NA			<18K: 4204 (31.01%)<br>18K-30.9K: 3646 (26.90%)<br>31K-51.9K: 3277 (24.17%)<br>52K-100K: 2064 (15.23%)<br>>100K: 365 (2.69%)		
	N missing	-			2793		

^ adjusted for age and sex

B

		EXCEED			UKB		
**Variable**	**Group**	**Description**	**Effect size**	**p**	**Description**	**Effect size**	**p**
Age at first diagnosis of depression^	TRD	40.89 (10.26)	OR=0.71 (0.60-0.84)	8.08e-5*	45.21 (11.16)	OR=0.88 (0.84-0.91)	1.09e-9*
	Non-TRD	44.57 (10.85)			46.72 (11.38)		
	N missing	0			13		
Age at first antidepressant prescription	TRD	42.16 (8.99)	OR=0.64 (0.53-0.76)	4.18e-7*	46.78 (8.64)	OR=0.70 (0.67-0.73)	3.08e-56*
	Non-TRD	46.19 (9.13)			50.02 (9.38)		
	N missing	0			0		
N of distinct antidepressants for ≥ 6 weeks	TRD	4.76 (1.55)	OR=7.68 (7.45-7.93)	<5e-324*	4.29 (1.49)	OR=11.40 (10.43-12.46)	<5e-324*
	Non-TRD	2.04 (0.95)			1.74 (0.89)		
	N missing	0			0		
N of antidepressants for ≥ 6 weeks	TRD	8.20 (3.30)	OR=1.82 (1.75-1.90)	6.57e-178*	6.50 (2.84)	OR=5.95 (5.58-6.35)	<5e-324*
	Non-TRD	3.94 (2.15)			2.58 (1.66)		
	N missing	0			0		
Proportion of adequate prescription intervals (≤14 weeks)	TRD	0.94 (0.09)	Kruskal-Wallis chi2=390	0.078	0.89 (0.09)	Kruskal-Wallis chi2=287	1.97e-64*
	Non-TRD	0.93 (0.10)			0.84 (0.14)		
	N missing	0			0		
≥ one overlap between year of diagnosis and year of antidepressant prescription (%)	TRD	158 (99.37%)	OR=9.76 (8.04-11.85)	1.94e-117*	2340 (96.30%)	OR=3.54 (2.86-4.40)	1.55e-30*
	Non-TRD	954 (93.53%)			14038 (88.01%)		
	N missing	0			0		
**Personality traits at baseline**							
“Are you often troubled by feelings of guilt?” (yes/total)	TRD	NA	NA	NA	1366/2350 (58%)	OR = 1.56 (1.43 – 1.71)	1.63e-23*
	Non-TRD	NA			7248/15419 (47%)		
	N missing	-			612		
“Do you often feel lonely?” (yes/total)	TRD	NA	NA	NA	1190/2369 (50.23%)	OR = 1.69 (1.55 – 1.84)	3.98e-32*
	Non-TRD	NA			5819/15543 (37.43%)		
	N missing	-			469		

"Are your feelings easily hurt?" (yes/total)	TRD	NA	NA	NA	1898/2365 (80.25%)	OR = 1.48 (1.33 – 1.64)	1.01e-12*
	Non-TRD	NA			11383/15521 (73.34%)		
	N missing	-			495		
"Do you ever feel 'just miserable' for no reason?" (yes/total)	TRD	NA	NA	NA	1984/2393 (82.91%)	OR = 1.91 (1.71 – 2.14)	8.72e-30*
	Non-TRD	NA			11227/15650 (71.74%)		
	N missing	-			338		
"Are you an irritable person?" (yes/total)	TRD	NA	NA	NA	1197/2297 (51.11%)	OR = 1.52 (1.39 – 1.66)	8.44e-21*
	Non-TRD	NA			6278/15060 (41.69%)		
	N missing	-			1024		
"Does your mood often go up and down?" (yes/total)	TRD	NA	NA	NA	2057/2397 (85.82%)	OR = 2.12 (1.88 – 2.40)	9.97e-35*
	Non-TRD	NA			11571/15635 (74.01%)		
	N missing	-			349		
"Would you call yourself a nervous person?" (yes/total)	TRD	NA	NA	NA	1201/2339 (51.35%)	OR = 1.68 (1.54 – 1.84)	1.64e-31*
	Non-TRD	NA			5930/15385 (38.54%)		
	N missing	-			657		
Neuroticism score	TRD	NA	NA	NA	7.97 (3.15)	OR = 1.59 (1.50 – 1.67)	3.00e-66*
	Non-TRD	NA			6.35 (3.30)		
	N missing	-			4089		
"Would you describe yourself as someone who takes risks?" (yes/total)	TRD	NA	NA	NA	585/2327 (25.14%)	OR = 0.95 (0.86 – 1.05)	0.32
	Non-TRD	NA			3982/15245 (26.12%)		
	N missing	-			809		

^ Values < 13 years were set to missing (n=13)

C

		EXCEED			UKB		
**Variable**	**Group**	**Description**	**Effect size**	**p**	**Description**	**Effect size**	**p**
BMI^	TRD	30.87 (7.14)	OR=1.26 (1.09-1.46)	0.002*	29.52 (5.98)	OR=1.26 (1.21-1.31)	1.29e-29*
	Non-TRD	28.83 (7.24)			28.16 (5.32)		
	N missing	53			123		
Obesity (BMI ≥ 30)^ (yes/total)	TRD	70/152 (46.05%)	OR=1.74 (1.23-2.47)	0.002*	973/2408 (40.41%)	OR=1.57 (1.43-1.71)	2.20e-23*
	Non-TRD	326/989 (32.96%)			4785/15850 (30.19%)		
	N missing	38			123		
Diabetes diagnosed by doctor^^ (yes/total)	TRD	25/159 (15.72%)	OR=2.07 (1.23-3.47)	0.006	190/2413 (7.87%)	OR=1.06 (0.89-1.25)	0.52
	Non-TRD	78/1020 (7.65%)			987/15865 (6.22%)		
	N missing	0			103		
Cardiovascular disease diagnosed by doctor^^ (yes/total)	TRD	8/159 (5.03%)	OR=1.68 (0.75-3.77)	0.21	896/2423 (36.98%)	OR=1.13 (1.02-1.24)	0.016
	Non-TRD	37/1020 (3.63%)			5014/15871 (31.59%)		
	N missing	0			87		
Cancer diagnosed by doctor^^ (yes/total)	TRD	6/159 (3.77%)	OR=0.82 (0.34-1.97)	0.66	239/2413 (9.90%)	OR=1.18 (1.02-1.36)	0.03
	Non-TRD	48/1020 (4.71%)			1338/15854 (8.44%)		
	N missing	0			114		
Longstanding illness or disability^^ (yes/total)	TRD	NA	NA	NA	1534/2333 (65.75%)	OR = 2.26 (2.06 – 2.49)	5.45e-64*
	Non-TRD	NA			6906/15409 (44.82%)		
	N missing	-			639		
Ever smoker (yes/total)	TRD	79/152 (51.97%)	OR=0.92 (0.66-1.30)	0.64	1213/2418 (50.17%)	OR=1.02 (0.93-1.11)	0.73
	Non-TRD	534/989 (53.99%)			7894/15854 (49.79%)		
	N missing	38			109		
Current smoker/never smoker	TRD	24/97	OR=1.23 (0.75-2.04)	0.41	372/1205	OR=1.05 (0.92-1.19)	0.48
	Non-TRD	121/575			2348/7960		
	N missing	38			109		
Alcohol intake^, as weekly units in EXCEED and alcohol intake frequency, N per group (%), in UKB	TRD	10.26 (19.64)	OR=0.97 (0.81-1.16)	0.71	Almost daily: 336 (13.84%)<br>3-4 week: 349 (14.37%)<br>1-2 week: 580 (23.89%)<br>1-3 month: 339 (13.96%)<br>Special occasions: 489 (20.14%)	Almost daily: OR=0.90 (0.78-1.04)<br><br>3-4 week: OR=0.82	0.15<br><br>5.74e-3

					Never: 335 (13.80%)	(0.71-0.94)<br><br>1-2 week: reference level<br><br>1-3 month: OR=1.11 (0.96-1.28)<br><br>Special occasions: OR=1.43 (1.25-1.63)<br><br>Never: OR=1.49 (1.28-1.72)	 <br><br><br>0.17<br><br><br>8.51e-8*<br><br><br>1.12e-7*
	Non-TRD	10.96 (18.05)			Almost daily: 2650 (16.66%)<br>3-4 week: 3026 (19.03%)<br>1-2 week: 4107 (25.82%)<br>1-3 month: 2166 (13.62%)<br>Special occasions: 2383 (14.98%)<br>Never: 1523 (9.89%)		
	N missing	41			48		
Days/week of moderate physical activity^^^	TRD	NA	NA	NA	3.33 (2.45)	OR = 0.92 (0.88 – 0.96)	1.39e-4*
	Non-TRD	NA			3.53 (2.40)		
	N missing	-			1276		

^ adjusted for age and sex. In UKB, after adjusting also for socio-economic status (household income and education), “special occasions” and “never” remained associated with TRD (p=1.27e-4, OR=1.33 [1.15-1.53], and p=2.03e-4, OR=1.37 [1.16-1.62], respectively)

^^ Adjusted for age, sex, BMI and ever smoker

^^^ adjusted for age and sex; no longer significant if adjusted also for long-term disability or illness (p=3.29e-3)

Supplementary Table 6: p-values (Fisher's test) of comparisons in the proportion of missing values in clinical-demographic variables between TRD and non-TRD groups in EXCEED and UKB. Only variables having missing values in at least one of two cohorts are shown. * Significant after Bonferroni correction (considering 26 independent variables tested in UKB, alpha=0.00192).

**Variable**	**EXCEED**	**UKB**
Age when attended assessment centre	0.34	No missing
Age at first diagnosis of depression	No missing	0.24
Ethnic background	0.34	0.76
Index of multiple deprivation quintile in EXCEED and townsend deprivation index in UKB	1	0.57
BMI	0.22	0.14
Obesity	0.34	0.14
Diabetes diagnosed by doctor	No missing	0.31
Cardiovascular disease diagnosed by doctor	No missing	0.20
Cancer diagnosed by doctor	No missing	0.58
Ever smoker	0.34	0.57
Current smoker/never smoker	0.34	0.57
Weekly alcohol intake in EXCEED and alcohol intake frequency as N per group in UKB	0.25	0.08
Number in household	Variable not available	0.43
Leisure - social activities	Variable not available	0.24
Able to confide	Variable not available	0.07
Frequency of visits from family/friends	Variable not available	0.34
Days/week of moderate physical activity	Variable not available	0.00031*
Longstanding illness or disability	Variable not available	0.14
Education	Variable not available	0.07
Average household income	Variable not available	0.08
"Are you often troubled by feelings of guilt?"	Variable not available	0.95
"Do you often feel lonely?"	Variable not available	0.95
"Are your feelings easily hurt?"	Variable not available	1
"Do you ever feel 'just miserable' for no reason?"	Variable not available	0.22
"Are you an irritable person?"	Variable not available	0.85
"Does your mood often go up and down?"	Variable not available	0.04
"Would you call yourself a nervous person?"	Variable not available	0.64
Neuroticism score	Variable not available	0.81
"Would you describe yourself as someone who takes risks?"	Variable not available	0.71

Supplementary Table 7: psychiatric comorbidities in patients with treatment-resistant depression (TRD) vs non-TRD according to primary care records in UK Biobank (UKB).

**Comorbidities**	**TRD** (n=2,430)	**Non-TRD** (n=15,951)	**Effect size**	**P value**
Anxiety disorders	997 (41.03%)	4290 (26.89%)	OR=1.89 (1.73-2.07)	1.25e-45
Depression with anxiety	904 (37.20%)	4950 (31.03%)	OR=1.32 (1.20-1.44)	1.30e-9
Obsessive-compulsive disorder	60 (2.47%)	132 (0.083%)	OR=3.03 (2.23-4.13)	1.69e-12
Somatoform disorders	195 (8.02%)	860 (5.39%)	OR=1.53 (1.30-1.80)	2.43e-7
Stress-related disorders	196 (8.07%)	1014 (6.36%)	OR=1.29 (1.10-1.52)	0.0016
Eating disorders	45 (1.85%)	127 (0.080%)	OR=2.35 (1.67-3.31)	1.02e-6
Sleep disorders	83 (3.42%)	304 (1.91%)	OR=1.82 (1.42-2.33)	1.93e-6
Personality disorders	55 (2.26%)	147 (0.092%)	OR=2.49 (1.82-3.40)	1.09e-8
Self-harm/suicidal behaviours	132 (5.43%)	438 (2.28%)	OR=2.03 (1.67-2.48)	2.99e-12

Supplementary Table 8: frequency of psychotropic drug combinations (**A**), type of antidepressant drug combinations (**B**) and combinations based on drug classes (**C**) in UKB. Antidepressant augmentation with any antipsychotic or a mood stabilizer among lithium, valproate, lamotrigine and pregabalin were considered in patients with TRD and non-TRD. Only combinations prescribed for longer than 30 days were considered. When considering specific drug or class combinations, only those prescribed to > 15 subjects were considered. Fisher's exact test was used for comparisons because of the small size of some of the groups. Bonferroni corrected p values was 2.38e-3 and 1.43e-3 for the comparisons in B and C, respectively.
* significant difference between TRD and non-TRD.

A

**Drug combination**	**TRD** (n=2430)	**Non-TRD** (n=15951)	**Statistics**
Antidepressant combination	1128 (46.42%)	1307 (8.19%)	OR=5.66 (5.17-6.21), p=4.03e-289*
Augmentation with antipsychotic	354 (14.57%)	576 (3.61%)	OR=4.03 (3.50-4.65), p=2.95e-75*
Augmentation with mood stabilizer	326 (13.42%)	406 (2.55%)	OR=5.27 (4.51-6.15), p=4.33e-90*

B

**Drug combination**	**TRD**	**Non-TRD**	**Statistics**
amitriptyline-citalopram	198 (17.55%)	266 (20.35%)	OR=0.86 (0.70-1.06), p=0.15
amitriptyline-fluoxetine	144 (12.77%)	245 (18.75%)	OR=0.68 (0.54-0.85), p=6.56e-4*
amitriptyline-mirtazapine	73 (6.47%)	49 (3.75%)	OR=1.73 (1.17-2.55), p=3.87e-3
amitriptyline-paroxetine	32 (2.84%)	39 (2.98%)	OR=0.95 (0.57-1.57), p=0.90
amitriptyline-duloxetine	16 (1.42%)	12 (0.92%)	OR=1.54 (0.68-3.59), p=0.26
amitriptyline-sertraline	112 (9.93%)	121 (9.26%)	OR=1.07 (0.81-1.42), p=0.63
amitriptyline-venlafaxine	58 (5.14%)	8 (3.67%)	OR=8.39 (3.97-20.44), p=4.69e-12*
citalopram-dosulepin	20 (1.77%)	7 (0.54%)	OR=3.31 (1.34-9.30), p=5.62e-3
citalopram-fluoxetine	60 (5.32%)	30 (2.30%)	OR=2.32 (1.46-3.75), p=1.51e-4*
citalopram-mirtazapine	50 (4.43%)	58 (4.44%)	OR=1 (0.66-1.50), p=1
citalopram-sertraline	41 (3.63%)	32 (2.45%)	OR=1.48 (0.91-2.45), p=0.12
citalopram-venlafaxine	16 (1.42%)	6 (0.05%)	OR=3.09 (1.14-9.67), p=0.017
fluoxetine-mirtazapine	33 (2.93%)	25 (1.91%)	OR=1.53 (0.88-2.70), p=0.14
fluoxetine-sertraline	49 (4.34%)	11 (0.084%)	OR=5.16 (2.63-11.06), p=4.83e-8*

mirtazapine-sertraline	46 (4.08%)	38 (2.91%)	OR=1.40 (0.89-2.23), p=0.15
mirtazapine-venlafaxine	82 (7.27%)	65 (4.97%)	OR=1.46 (1.03-2.08), p=0.027
trazodone-citalopram	13 (1.15%)	18 (1.38%)	OR=0.84 (0.38-1.82), p=0.72
trazodone-fluoxetine	14 (1.24%)	19 (1.45%)	OR=0.85 (0.39-1.81), p=0.73
trazodone-venlafaxine	16 (1.42%)	12 (0.092%)	OR=1.54 (0.68-3.59), p=0.26
venlafaxine-citalopram	16 (1.42%)	12 (0.092%)	OR=1.54 (0.68-3.59), p=0.26
venlafaxine-sertraline	16 (1.42%)	3 (0.022%)	OR=6.17 (1.76-33.17), p=9.35e-4*

C

**Drug combination**	**TRD**	**Non-TRD**	**Statistics**
SNRI-NaSSA	97 (5.93%)	64 (4.45%)	OR=1.33 (0.95-1.87), p=0.09
SNRI-SARI	29 (1.78%)	23 (1.60%)	OR=1.11 (0.62-2.01), p=0.78
SNRI-SNRI	16 (0.98%)	2 (0.14%)	OR=7.09 (1.65-63.08), p=0.0031
SSRI-NaSSA	165 (10.08%)	128 (8.91%)	OR=1.13 (0.88-1.45), p=0.33
SSRI-phenothiazine	19 (1.16%)	8 (0.06%)	OR=2.08 (0.87-5.52), p=0.08
SSRI-SARI	70 (4.28%)	68 (4.73%)	OR=0.90 (0.63-1.29), p=0.60
SSRI-SNRI	126 (7.70%)	59 (4.11%)	OR=1.87 (1.35-2.62), p=7.33e-5*
SSRI-SSRI	195 (11.91%)	105 (7.31%)	OR=1.63 (1.27-2.11), p=9.75e-5*
TCA-NaSSA	94 (5.74%)	63 (4.38%)	OR=1.31 (0.93-1.85), p=0.12
TCA-SARI	41 (2.50%)	26 (1.81%)	OR=1.38 (0.82-2.37), p=0.22
TCA-SNRI	119 (7.27%)	99 (6.89%)	OR=1.06 (0.79-1.41), p=0.73
TCA-SSRI	594 (36.29%)	760 (52.89%)	OR=0.69 (0.60-0.78), p=9.17e-9*
TCA-TCA	72 (4.40%)	32 (2.23%)	OR=1.97 (1.28-3.12), p=0.0013*
NaSSA-atypical AP	63 (10.29%)	75 (9.99%)	OR=1.03 (0.71-1.49), p=0.93
NaSSA-typical AP	26 (4.25%)	29 (3.86%)	OR=1.10 (0.62-1.96), p=0.78
SARI-atypical AP	20 (3.27%)	15 (2.0%)	OR=1.64 (0.79-3.47), p=0.17
SARI-typical AP	26 (4.25%)	17 (2.26%)	OR=1.88 (0.97-3.72), p=0.06
SNRI-atypical AP	66 (10.78%)	84 (11.19%)	OR=0.96 (0.68-1.37), p=0.96
SNRI-typical AP	49 (8.01%)	46 (6.13%)	OR=1.31 (0.84-2.03), p=0.24
SSRI-atypical AP	97 (15.85%)	124 (16.51%)	OR=0.96 (0.71-1.29), p=0.83
SSRI-typical AP	131 (21.41%)	215 (28.63%)	OR=0.75 (0.58-0.96), p=0.021
TCA atypical AP	40 (6.54%)	35 (4.60%)	OR=1.40 (0.86-2.30), p=0.16
TCA typical AP	94 (15.36%)	111 (14.78%)	OR=0.82 (0.76-1.41), p=0.82
NaSSA-lithium	31 (6.11%)	18 (3.11%)	OR=1.97 (1.05-3.78), p=0.028
NaSSA-pregabalin	46 (9.07%)	28 (4.84%)	OR=1.88 (1.13-3.16), p=0.011

SARI-lithium	16 (3.16%)	8 (1.38%)	OR=2.28 (0.91-6.21), p=0.06
SNRI-lithium	46 (9.07%)	36 (6.22%)	OR=1.46 (0.91-2.36), p=0.11
SNRI-pregabalin	47 (9.27%)	43 (7.43%)	OR=1.25 (0.79-1.97), p=0.32
SSRI-lamotrigine	23 (4.54%)	15 (2.59%)	OR=1.75 (0.86-3.65), p=0.10
SSRI-lithium	61 (12.03%)	27 (4.66%)	OR=2.58 (1.59-4.29), p= 5.31e-5*
SSRI-pregabalin	134 (26.43%)	148 (25.56%)	OR=1.03 (0.79-1.36), p=0.84
SSRI-valproate	27 (5.33%)	25 (4.32%)	OR=1.23 (0.68-2.25), p=0.48
TCA-lithium	43 (8.48%)	32 (5.53%)	OR=1.53 (0.93-2.55), p=0.09
TCA-pregabalin	84 (16.57%)	119 (2.06%)	OR=0.80 (0.59-1.10), p=0.17
TCA-valproate	21 (4.12%)	8 (1.38%)	OR=2.99 (1.26-7.89), p=0.0076

Supplementary Table 9: description reported as median and interquartile range (in parenthesis) of the number of all clinical records (including diagnoses, symptoms, lab results, procedures) and all medication prescriptions in TRD and non-TRD groups in UK Biobank (UKB) and EXCEED. Records from 1985 forwards were considered as previous records were sparse. The p-values were obtained using a Kruskal-Wallis test. Plots describing these data are in Supplementary Figure 7. The lower number of clinical records in EXCEED than UKB likely reflects differences in the type of administrative codes that were extracted (e.g., referrals, screening invitations, issued certificates).

	**UK Biobank**			**EXCEED**		
**Variable**	**TRD**	**Non-TRD**	**p-value**	**TRD**	**Non-TRD**	**p-value**
Total number of clinical records	860.5 (655.75)	598.0 (534)	1.44e-65	519 (442)	344 (305)	2.67e-12
Number of clinical records registered on different dates	235 (168.75)	160.0 (127)	4.50e-130	161 (133.5)	110 (65)	5.82e-15
Time span of clinical records (years)	28 (5)	28 (5)	0.58	29 (6)	29 (6)	0.89
Total number of clinical records / time span of clinical records (years)	31.68 (24.81)	22.39 (20.05)	4.66e-12	19.15 (15.38)	12.53 (10.51)	2.08e-12
Number of clinical records registered on different dates / time span of clinical records (years)	8.73 (6.18)	6.04 (6.18)	5.45e-54	5.90 (4.12)	4 (3)	3.33e-16
Average time between consecutive clinical records (weeks)	6.01 (4.33)	8.71 (6.85)	0.066^	9.02 (6.17)	13.20 (9.91)	3.57e-16
Total number of prescription records	551.5 (683.00)	228 (393.5)	5.61e-175	563 (760)	191 (330.75)	4.11e-26
Number of prescription records registered on different dates	241 (182.75)	133 (147)	4.48e-237	241 (163.5)	125 (128)	5.82e-29
Time span of prescription records (years)	21 (8)	19 (9)	6.96e-38	20 (7)	19 (7)	2.08e-3
Total number of prescription records / time span of prescription records (years)	28.21 (34.37)	13.64 (22.81)	5.54e-66	29.35 (37.64)	11.30 (18.73)	2.81e-22

Number of prescription records registered on different dates / time span of prescription records (years)	12.38 (8.29)	8 (7.65)	6.93e-96	12.79 (8.45)	7.19 (6.84)	1.44e-24
Average time between consecutive prescription records (weeks)	4.22 (2.92)	6.54 (6.98)	0.24^	4.13 (2.90)	7.29 (7.88)	9.15e-25

^ In UKB there was a particularly long tail for this variable (see also Supplementary Figure 7), therefore the Kruskal-Wallis test provided a non-significant p-value; however, after log-transformation of the variable, a t-test showed a significant difference between TRD and non-TRD in terms of time between consecutive clinical records (p=1.15e-203) and time between consecutive prescription records (p=7.74e-275), in line with the significant difference found in EXCEED.

Supplementary Table 10: variants with p < 1e-5 for the comparison treatment-resistant depression (TRD) vs non-TRD in UKB. Positions (pos) are reported according to GRCh37. Variant annotation was performed using FUMA (https://fuma.ctglab.nl). Chr=chromosome; EA=effect allele; dist=distance; func=functional annotation.

Variant ID	chr	pos	EA	EA freq	beta	SE	p	INFO	nearest gene	dist	func	CADD	RDB
rs752299813	3	137186963	T	0.054	0.042	0.009	9.82E-07	0.999	RNA5SP142	50012	intergenic	8.116	NA
rs73863961	3	137122575	T	0.056	0.042	0.008	9.94E-07	0.998	RNA5SP142	114400	intergenic	2.476	6
rs140192346	1	234967410	GC	0.015	0.040	0.008	1.01E-06	0.944	RNY4P16	6309	intergenic	0	NA
rs755232584	3	137188987	A	0.054	0.042	0.009	1.07E-06	0.999	RNA5SP142	47988	intergenic	2.325	NA
rs13078081	3	66852901	C	0.243	0.038	0.008	1.09E-06	0.998	RPL21P41	165064	intergenic	3.219	6
rs58350491	3	137187721	G	0.054	0.042	0.009	1.14E-06	0.999	RNA5SP142	49254	intergenic	0	7
rs4536769	3	137195633	G	0.054	0.042	0.009	1.16E-06	0.999	RNA5SP142	41342	intergenic	1.789	7
rs147270579	20	338460	G	0.085	0.037	0.008	1.34E-06	0.999	NRSN2	0	intronic	12,1	6
rs12490164	3	137196162	C	0.054	0.041	0.009	1.34E-06	0.999	RNA5SP142	40813	intergenic	8.806	7
rs12489530	3	137199215	T	0.056	0.042	0.009	1.45E-06	0.995	RNA5SP142	37760	intergenic	1.339	6
rs147081653	20	338111	A	0.085	0.037	0.008	1.45E-06	0.999	NRSN2	0	intronic	0,04	6
rs144495474	11	30232200	C	0.018	0.037	0.008	1.69E-06	0.998	FSHB	20362	intergenic	3.929	3a
rs59120530	3	137181392	G	0.053	0.040	0.008	1.82E-06	1.000	RNA5SP142	55583	intergenic	1.201	5
rs4362212	12	66948167	C	0.543	-0.037	0.008	2.03E-06	1.000	GRIP1	0	intronic	0,66	7
rs78298378	20	344195	C	0.086	0.037	0.008	2.07E-06	0.998	NRSN2	3890	intergenic	2.704	3a
rs12490219	3	137196378	C	0.053	0.040	0.008	2.13E-06	0.999	RNA5SP142	40597	intergenic	20,1	6
rs78339301	11	30301746	G	0.018	0.036	0.008	2.14E-06	0.997	ARL14EP	42851	intergenic	1.223	5
rs76686854	20	342463	T	0.086	0.037	0.008	2.14E-06	1.000	NRSN2	2158	intergenic	2.618	4
20:342643_TA_T	20	342643	T	0.086	0.037	0.008	2.20E-06	1.000	NRSN2	2338	intergenic	2.855	NA
rs6565595	17	79521649	C	0.745	0.037	0.008	2.31E-06	0.999	C17orf70	661	upstream	1.766	5
rs564958966	2	81789701	T	0.047	0.037	0.008	2.35E-06	0.964	RNA5SP99	66249	intergenic	3.191	NA
rs571956382	2	81789702	A	0.046	0.037	0.008	2.37E-06	0.963	RNA5SP99	66250	intergenic	3.213	NA
rs62153281	2	81737394	T	0.062	0.037	0.008	2.41E-06	0.995	RNA5SP99	13942	intergenic	1	7
rs2131596	12	66952640	G	0.548	-0.037	0.008	2.54E-06	0.999	GRIP1	0	intronic	2,45	NA
rs115633075	3	137198107	T	0.056	0.040	0.009	2.62E-06	0.994	RNA5SP142	38868	intergenic	0,32	6

rs7633165	3	137180994	C	0.056	0.041	0.009	2.83E-06	0.999	RNA5SP142	55981	intergenic	0	7
rs1476777073	2	137056768	CTTCT	0.011	0.039	0.008	2.92E-06	0.991	UBBP1	30102	intergenic	NA	NA
rs1493489	12	66944708	G	0.545	-0.037	0.008	2.95E-06	0.999	GRIP1	0	intronic	1.974	NA
rs565448776	18	36493938	A	0.015	0.035	0.007	2.99E-06	0.931	RN7SKP182	102072	intergenic	3,04	NA
rs4476458	3	137148949	C	0.055	0.040	0.009	3.34E-06	0.998	RNA5SP142	88026	intergenic	4.345	6
rs7207933	17	79521239	G	0.745	0.037	0.008	3.36E-06	0.999	C17orf70	251	upstream	5.259	1b
rs199707301	3	137174197	A	0.048	0.038	0.008	3.38E-06	0.997	RNA5SP142	62778	intergenic	1.736	7
rs370542060	3	137174129	C	0.048	0.038	0.008	3.39E-06	0.997	RNA5SP142	62846	intergenic	2.251	6
rs56093609	6	21095911	G	0.045	0.036	0.008	3.46E-06	0.994	CDKAL1	0	intronic	3.902	5
rs186910289	2	137056724	C	0.010	0.038	0.008	3.53E-06	0.995	UBBP1	30286	intergenic	0,48	6
rs61904805	11	116433091	A	0.010	-0.037	0.008	3.54E-06	0.977	AP001891,1	61743	intergenic	14,7	5
rs776682966	3	137156035	G	0.055	0.039	0.009	3.68E-06	0.997	RNA5SP142	80940	intergenic	9.223	NA
rs116949009	8	140040373	T	0.018	0.038	0.008	3.79E-06	1.000	RP11-324F11,1	68174	intergenic	0	5
rs1147165	5	92249816	C	0.772	0.036	0.008	3.87E-06	0.999	CTC-458G6,2	0	ncRNA_exonic	0	NA
4:151699124_CAAA_C	4	151699124	C	0.729	0.037	0.008	4.05E-06	0.979	LRBA	0	intronic	1.234	NA
2:137053739_CT_C	2	137053739	C	0.010	0.038	0.008	4.06E-06	0.997	UBBP1	33271	intergenic	1.316	NA
rs116979514	11	45219710	T	0.017	0.035	0.007	4.15E-06	0.991	PRDM11	0	intronic	7.201	5
rs4960705	7	154455403	G	0.764	0.037	0.008	4.16E-06	0.949	DPP6	0	intronic	1,85	7
rs4396827	3	137141316	C	0.054	0.039	0.009	4.23E-06	0.999	RNA5SP142	95659	intergenic	0,45	6
rs56691960	1	9358445	A	0.130	-0.035	0.008	4.24E-06	0.935	SPSB1	0	intronic	1.173	NA
rs201721436	8	93517371	T	0.067	0.036	0.008	4.43E-06	0.909	LOC105375639	0	intronic	0	NA
rs12485887	3	137125982	C	0.054	0.039	0.009	4.57E-06	0.999	RNA5SP142	110993	intergenic	19	7
3:137147143_TA_T	3	137147143	T	0.050	0.038	0.008	4.62E-06	0.998	RNA5SP142	89832	intergenic	0	NA
rs4342059	3	137168088	A	0.054	0.039	0.008	4.63E-06	0.999	RNA5SP142	68887	intergenic	12,7	7
rs350802	5	92246357	T	0.772	0.035	0.008	4.66E-06	1.000	CTC-458G6,2	0	ncRNA_intronic	4.737	NA
rs79834175	5	172419037	T	0.029	0.038	0.008	4.72E-06	0.946	ATP6V0E1	0	NA	0,05	7
rs187057	5	92247474	A	0.772	0.035	0.008	5.06E-06	1.000	CTC-458G6,2	0	ncRNA_intronic	4.609	NA
rs350803	5	92246983	C	0.772	0.035	0.008	5.12E-06	1.000	CTC-458G6,2	0	ncRNA_intronic	1	NA
rs76262309	2	137050693	C	0.010	0.037	0.008	5.20E-06	0.998	UBBP1	36317	intergenic	0	5

rs350800	5	92242968	T	0.771	0.035	0.008	5.27E-06	0.999	CTC-458G6,2	0	ncRNA_exonic	5.574	4
rs350801	5	92243511	A	0.771	0.035	0.008	5.52E-06	1.000	CTC-458G6,2	0	ncRNA_intronic	4.135	7
rs140030682	1	168250879	T	0.011	0.034	0.008	5.53E-06	0.986	TBX19	0	intronic	6.328	4
rs149457990	4	27495358	AG	0.063	0.035	0.008	5.54E-06	0.996	IGBP1P5	91408	intergenic	0,91	NA
rs7431514	3	137134979	C	0.054	0.039	0.009	5.55E-06	0.998	RNA5SP142	101996	intergenic	5.202	6
rs11685088	2	137054094	C	0.010	0.037	0.008	5.57E-06	0.998	UBBP1	32916	intergenic	0	7
rs11683082	2	137048639	G	0.010	0.037	0.008	5.58E-06	0.997	UBBP1	38371	intergenic	1.537	6
rs7213717	17	79521181	T	0.743	0.036	0.008	5.65E-06	0.999	C17orf70	193	upstream	6.544	4
rs55985402	17	71831784	A	0.134	0.035	0.008	5.78E-06	0.984	CTD-2532D12,5	0	ncRNA_intronic	9.436	4
rs55821194	21	45146374	A	0.043	0.034	0.007	6.10E-06	0.992	PDXK	0	intronic	2.857	7
rs11117364	16	88132199	G	0.675	-0.035	0.008	6.18E-06	0.997	RP11-863P13,4	0	ncRNA_intronic	1.926	2b
rs117136231	20	60726778	A	0.010	0.034	0.007	6.24E-06	0.983	SS18L1	0	intronic	1	7
rs74815160	17	29157158	T	0.211	0.035	0.008	6.61E-06	0.989	CTD-2349P21,1	760	upstream	1	6
rs113246227	22	35849426	T	0.021	0.035	0.008	6.80E-06	0.926	MCM5	0	intronic	1.966	NA
rs78546238	15	24243084	C	0.019	0.033	0.007	7.62E-06	0.976	PWRN4	0	ncRNA_intronic	2.741	5
rs4519713	3	174534205	C	0.143	0.037	0.008	7.64E-06	0.918	NAALADL2	0	intronic	0	6
rs782174599	1	206490017	T	0.509	-0.035	0.008	7.95E-06	0.892	RP11-421E17,4	16660	intergenic	1.448	NA
rs8010048	14	31189112	A	0.017	0.038	0.008	7.98E-06	1.000	SCFD1	0	intronic	1.986	6
rs117497020	6	91458538	C	0.012	0.034	0.008	8.03E-06	0.993	MAP3K7	161773	intergenic	1.406	6
rs77346794	2	137052712	A	0.012	0.040	0.009	8.27E-06	1.000	UBBP1	34298	intergenic	1.759	6
rs1769181	1	239983734	G	0.387	0.035	0.008	8.40E-06	0.998	CHRM3	0	intronic	0	7
rs1934349	1	239984925	G	0.599	-0.035	0.008	9.05E-06	1.000	CHRM3	0	intronic	0,05	7
rs6429160	1	239984560	C	0.598	-0.035	0.008	9.07E-06	1.000	CHRM3	0	intronic	1	6
rs76617142	3	18820324	G	0.022	0.035	0.008	9.15E-06	1.000	AC144521,1	0	ncRNA_intronic	3.305	5
rs4463655	1	239984294	C	0.598	-0.035	0.008	9.16E-06	1.000	CHRM3	0	intronic	8.165	6
rs74530133	17	79516050	T	0.772	0.035	0.008	9.25E-06	0.999	C17orf70	0	intronic	4.533	7
rs569128897	2	200057544	C	0.016	0.036	0.008	9.46E-06	0.976	RNU7-147P	1700	intergenic	1	NA
rs575490301	6	7037168	CT	0.083	0.034	0.008	9.49E-06	0.964	snoU13	3524	intergenic	7.547	NA
rs148016647	11	57145026	GT	0.021	0.034	0.008	9.65E-06	0.987	PRG3	0	intronic	6.738	NA

Supplementary Table 11: genetic correlations (Rg) between TRD and non-TRD compared to healthy controls and TRD compared to non-TRD in UKB. Traits with rg with $p < 0.05$ for the comparison TRD vs. non-TRD are in bold. These results are represented in Supplementary Figure 8.

	**TRD vs controls**			**non-TRD vs controls**			**TRD vs non-TRD**		
	Rg	SE	p	Rg	SE	p	Rg	SE	p
**Major depressive disorder**	0.70	0.08	8.81e-17	0.69	0.05	7.31e-37	0.33	0.11	0.0028
**Depressive symptoms**	0.86	0.09	1.54e-19	0.87	0.07	3.84e-38	0.34	0.15	0.029
**Bipolar disorder**	0.28	0.06	7.68e-6	0.20	0.04	8.45e-6	0.21	0.09	0.024
**Schizophrenia**	0.35	0.06	1.21e-8	0.23	0.04	2.14e-8	0.26	0.10	0.0077
**ADHD**	0.69	0.24	0.0041	0.25	0.16	0.11	0.86	0.39	0.030
ASD	0.08	0.10	0.40	-0.03	0.07	0.65	0.15	0.14	0.28
Anorexia nervosa	0.14	0.07	0.06	0.12	0.06	0.029	0.08	0.11	0.47
PGC cross-disorder study	0.51	0.11	4.13e-6	0.32	0.06	7.01e-7	0.44	0.25	0.073
Neuroticism	0.71	0.08	2.63e-18	0.73	0.05	1.97e-42	0.23	0.12	0.054
Openness to experience	-0.04	0.17	0.82	-0.07	0.12	0.55	0.03	0.25	0.91
Consciousness	-0.26	0.22	0.22	-0.19	0.15	0.22	-0.14	0.32	0.67
**Subjective wellbeing**	-0.57	0.11	5.90e-7	-0.52	0.07	1.95e-14	-0.31	0.15	0.039
**Insomnia**	0.50	0.09	7.34e-8	0.40	0.06	1.43e-10	0.28	0.12	0.024
Sleep duration	-0.13	0.09	0.17	-0.07	0.07	0.32	-0.07	0.13	0.57
Chronotype (morningness)	-0.06	0.07	0.38	-0.02	0.05	0.69	-0.10	0.10	0.30
**Childhood IQ**	-0.43	0.13	0.0011	-0.20	0.11	0.056	-0.44	0.22	0.043
**Intelligence**	-0.41	0.06	8.02e-13	-0.36	0.04	9.99e-22	-0.20	0.08	0.0085
Years of schooling	-0.34	0.05	3.85e-12	-0.42	0.04	5.08e-31	-0.04	0.06	0.52
College completion	-0.43	0.08	3.12e-7	-0.49	0.07	2.22e-13	-0.12	0.12	0.29
LDL cholesterol	0.03	0.08	0.68	-0.07	0.06	0.28	0.14	0.12	0.24
HDL cholesterol	-1.56e-05	0.07	0.99	-0.07	0.06	0.23	0.01	0.10	0.90
Triglycerides	0.20	0.06	9.0e-4	0.12	0.05	0.019	0.17	0.10	0.087
Type 2 diabetes	0.05	0.09	0.57	0.03	0.07	0.62	0.11	0.12	0.37
Coronary artery disease	0.29	0.07	6.21e-5	0.27	0.05	2.34e-7	0.17	0.09	0.078
Ever smoked	0.34	0.10	8.0e-4	0.41	0.07	3.49e-9	0.02	0.12	0.86

Obesity class 1	0.10	0.06	0.12	0.16	0.05	4.0e-4	-0.03	0.10	0.73
Obesity class 2	0.06	0.08	0.46	0.15	0.06	0.0086	-0.06	0.14	0.63
Obesity class 3	0.32	0.11	0.004	0.27	0.08	7.0e-4	0.26	0.25	0.30
BMI	0.12	0.06	0.0418	0.18	0.04	3.37e-5	-0.02	0.08	0.83
Waist-to-hip ratio	0.20	0.06	0.0012	0.23	0.04	1.36e-7	0.04	0.09	0.68

Supplementary Table 12: estimation of power for polygenic risk score (PRS) analysis. We used the R package avengeme; P_T showing adequate power (≥0.80) are highlighted in green. P_T = p-value threshold. *cov* = covariance between genetic effect sizes in the base and target samples. ADHD=attention-deficit hyperactivity disorder. SCZ=schizophrenia. BP=bipolar disorder. NEU=neuroticism. SUBWB=subjective wellbeing. Child. IQ=childhood IQ.

Base trait	h^2_{SNP} base trait	*cov*	P_T	Power
MDD	0.072	0.025	5e-8	1
			1e-5	1
			1e-3	1
			0.01	1
			0.05	1
			0.1	0.97
			0.2	0.63
			0.3	0.36
			0.4	0.23
			0.5	0.17
			1	0.07
ADHD	0.240	0.119	5e-8	1
			1e-5	1
			1e-3	1
			0.01	1
			0.05	1
			0.1	1
			0.2	1
			0.3	1
			0.4	1
			0.5	1
			1	1
SCZ	0.402	0.072	5e-8	1
			1e-5	1
			1e-3	1
			0.01	1
			0.05	1
			0.1	1
			0.2	1
			0.3	1
			0.4	1
			0.5	1
			1	1
BP	0.347	0.035	5e-8	1
			1e-5	1
			1e-3	1
			0.01	1
			0.05	0.99
			0.1	0.93

			0.2	0.70
			0.3	0.48
			0.4	0.34
			0.5	0.26
			1	0.12
NEU	0.150	0.025	1e-3	1
			0.01	1
			0.05	0.98
			0.1	0.82
			0.2	0.39
			0.3	0.20
			0.4	0.13
			0.5	0.09
			1	0.04
SUBWB	0.025	0.014	5e-8	1
			1e-5	1
			1e-3	1
			0.01	1
			0.05	1
			0.1	1
			0.2	0.85
			0.3	0.63
			0.4	0.46
			0.5	0.35
			1	0.17
Intelligence	0.191	0.026	5e-8	0.92
			1e-5	0.92
			1e-3	0.92
			0.01	0.91
			0.05	0.84
			0.1	0.73
			0.2	0.50
			0.3	0.35
			0.4	0.26
			0.5	0.20
			1	0.11
Child. IQ	0.276	0.065	1e-5	1
			1e-3	1
			0.01	1
			0.05	1
			0.1	1
			0.2	1
			0.3	0.99
			0.4	0.95
			0.5	0.88
			1	0.57

Supplementary Table 13: **A.** results of the association analyses between the PRS of interest and the risk of treatment-resistant depression (TRD) vs. non-TRD in UKB. PRSs were standardized for easier interpretability. Nagelkerke R2 is reported on the observed scale since the comparison is referred to cases only. *significant results after Bonferroni correction (p=5.88e-4). P_T= p threshold, ADHD= Attention-deficit hyperactivity disorder. **B.** Results of multivariate regression models including ADHD PRS and each of the nominally significant PRS found in A.

A

**Base data**	**Best P_T**	**E (SE)**	**P**	**OR (95% CI)**	**Nagelkerke R2**
Major depressive disorder	0.001	0.05 (0.02)	0.028	1.05 (1.01-1.10)	5.36e-4
ADHD	0.2	0.08 (0.02)	4.38e-4*	1.09 (1.04-1.14)	1.38e-3
Schizophrenia	0.1	0.04 (0.02)	0.14	1.04 (0.99-1.09)	2.48e-4
Bipolar disorder	0.01	0.04 (0.02)	0.07	1.04 (0.99-1.09)	3.74e-4
Neuroticism	0.01	0.06 (0.02)	7.42e-3	1.06 (1.02-1.11)	7.98e-4
Subjective well-being	0.2	-0.08 (0.03)	4.64e-3	0.93 (0.88-0.98)	1.20e-3
Intelligence	0.1	-0.09 (0.03)	4.61e-3	0.91 (0.86-0.97)	1.50e-3
Childhood IQ	0.01	-0.02 (0.02)	0.29	0.98 (0.93-1.02)	1.25e-4

B

**Model**	**E (SE)**	**P**	**OR (95% CI)**	**Nagelkerke R2**
ADHD PRS with neuroticism PRS as covariate	0.08 (0.02)	4.51e-4*	1.09 (1.04-1.14)	2.17e-3
ADHD PRS with subjective well-being PRS as covariate	0.08 (0.02)	3.15e-3	1.08 (1.03-1.14)	2.51e-3
ADHD PRS with intelligence PRS as covariate	0.06 (0.03)	0.041	1.06 (1.002-1.13)	2.28e-3

Supplementary Figure 1: number of diagnostic codes for depressive disorders (codes reported > 500 times between 1990 and 2015 were represented; diagnosis descriptors were grouped in the represented categories) (**A**) and number of individuals receiving antidepressant prescriptions by drug (only drugs prescribed to > 1000 individuals) (**B**) and drug class (**C**) in UKB. To provide a comprehensive picture, these figures include all participants with at least one diagnostic code for depression and no diagnostic code for bipolar disorders, psychotic disorders or substance use disorders (n=36,880). Note that the number of diagnostic records per year tend to decrease after 2006, while the number of prescription records increases in time; this is due to the fact that diagnostic codes were usually not repeated in different years.

A

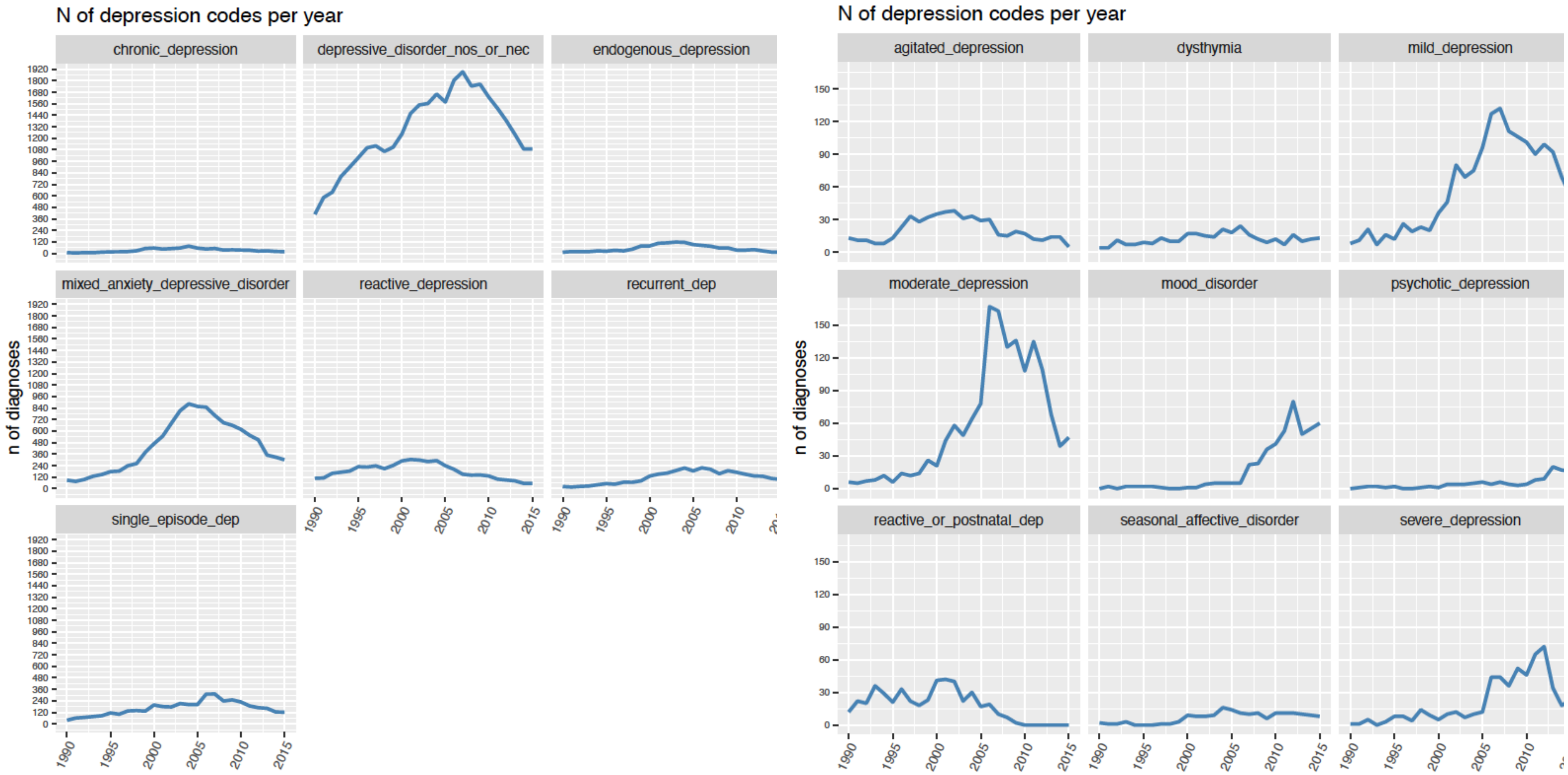

B

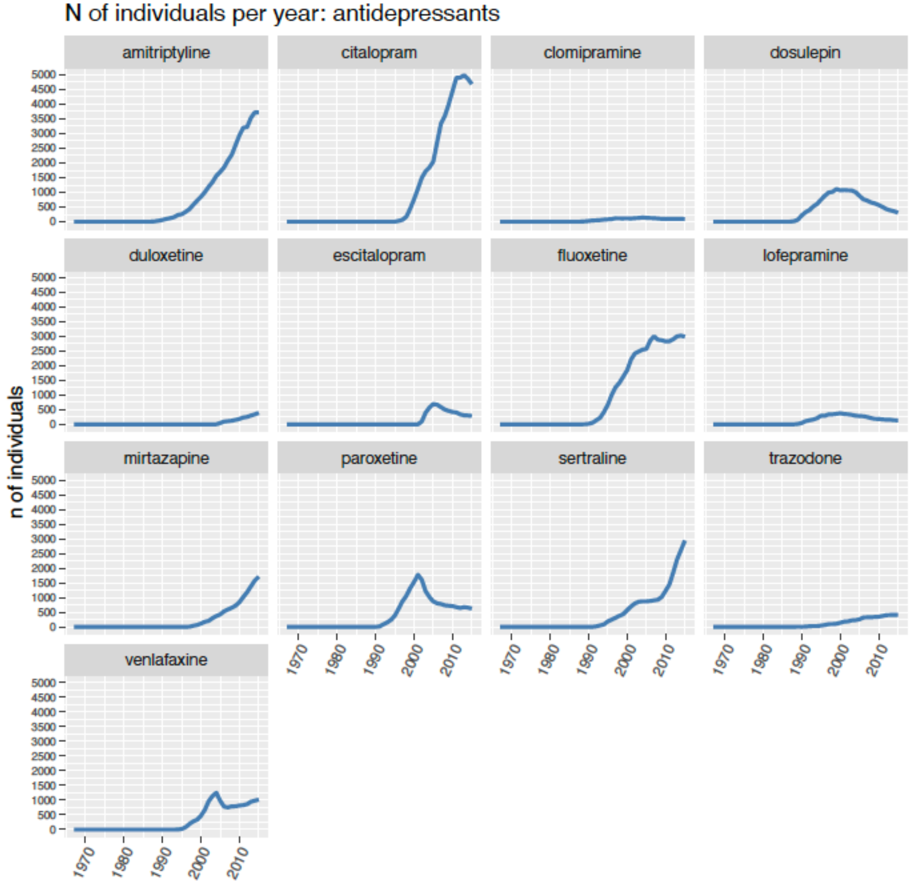

N of individuals per year: antidepressants
amitriptyline
citalopram
clomipramine
dosulepin
duloxetine
escitalopram
fluoxetine
lofepramine
mirtazapine
paroxetine
sertraline
trazodone
venlafaxine
n of individuals

C

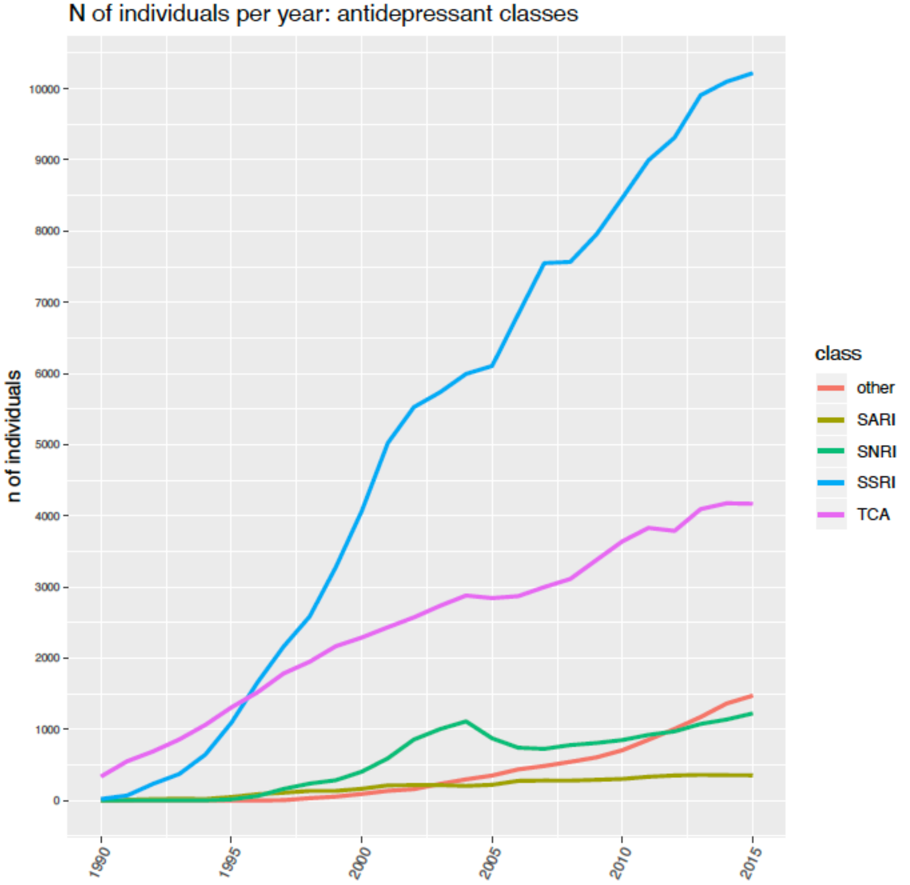

N of individuals per year: antidepressant classes
n of individuals
class
other
SARI
SNRI
SSRI
TCA

Supplementary Figure 2: representation of the townsend social deprivation index at recruitment in UKB. Each participant is assigned a score corresponding to the output area in which their postcode is located. Higher scores indicate higher social deprivation.

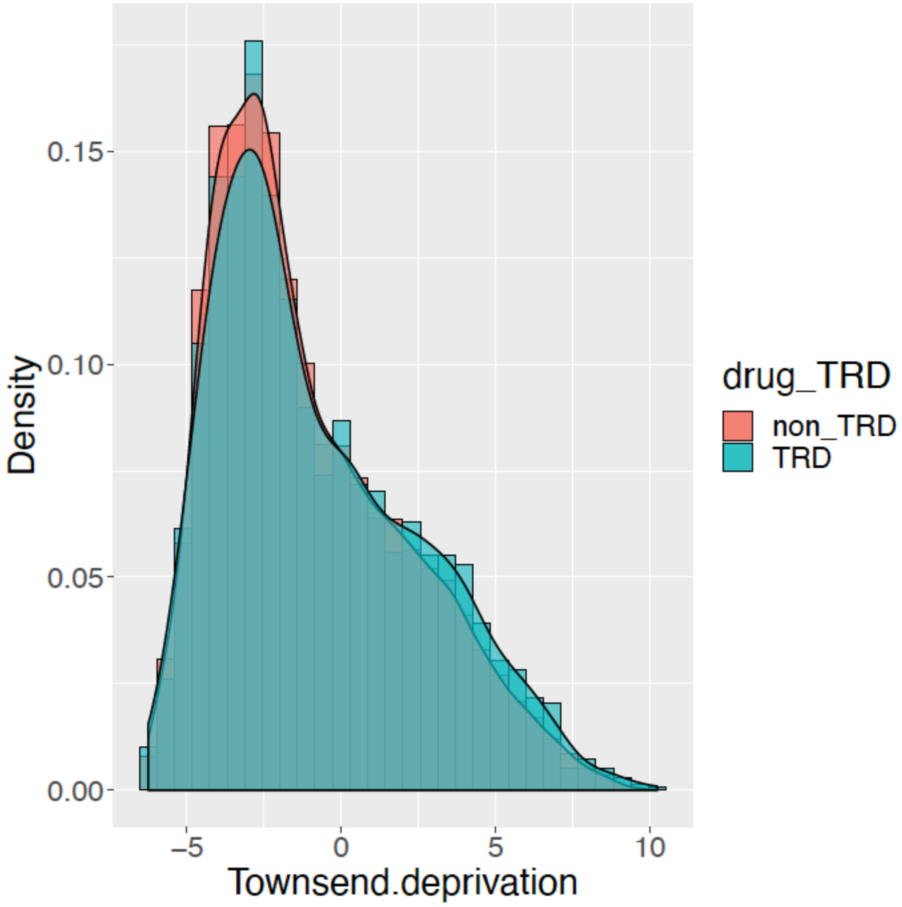

Supplementary Figure 3: distribution of education (**A**) and average household income (£) (**B**) categories in people with TRD vs non-TRD in UKB.

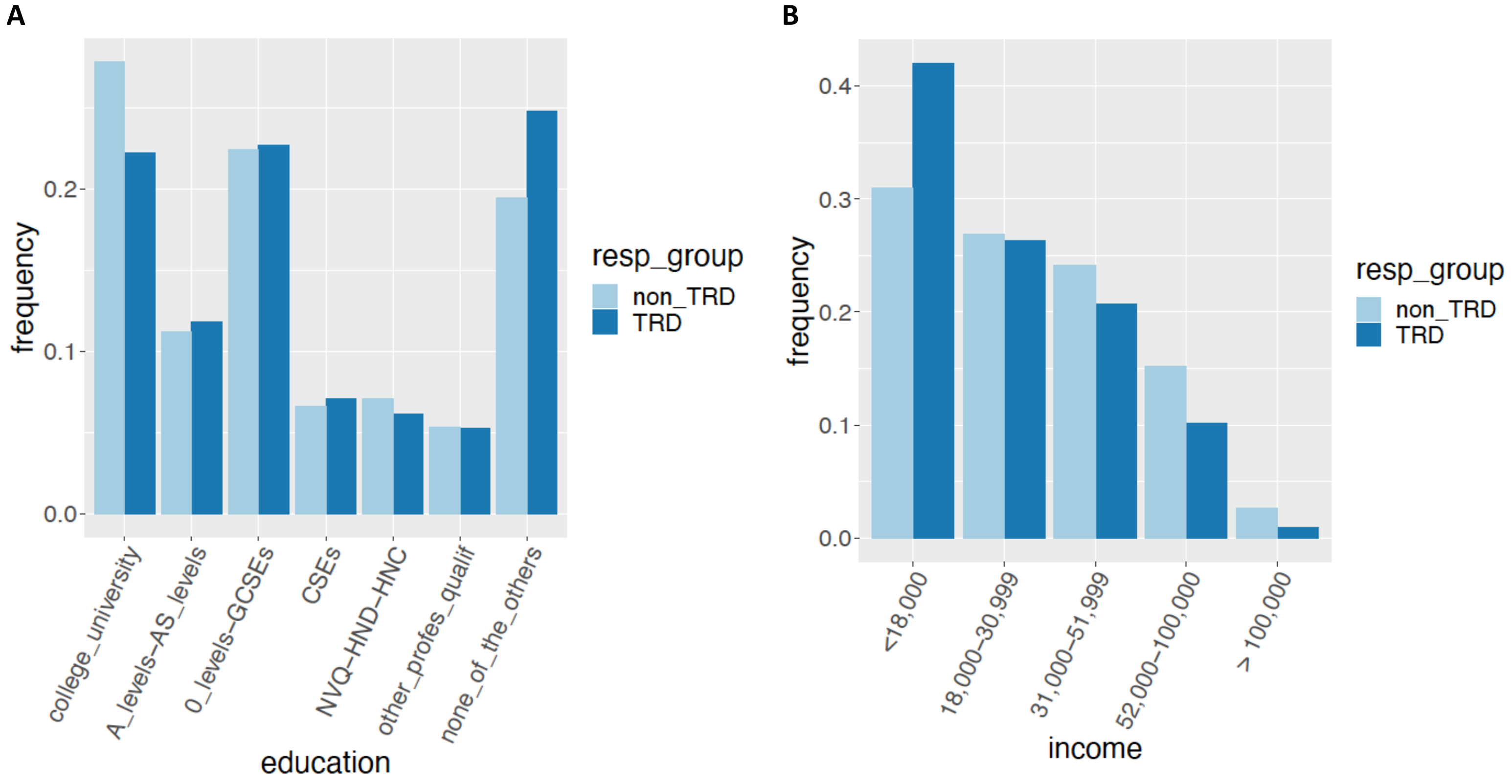

Supplementary Figure 4: frequency of alcohol intake in people with TRD vs. non-TRD in UKB.

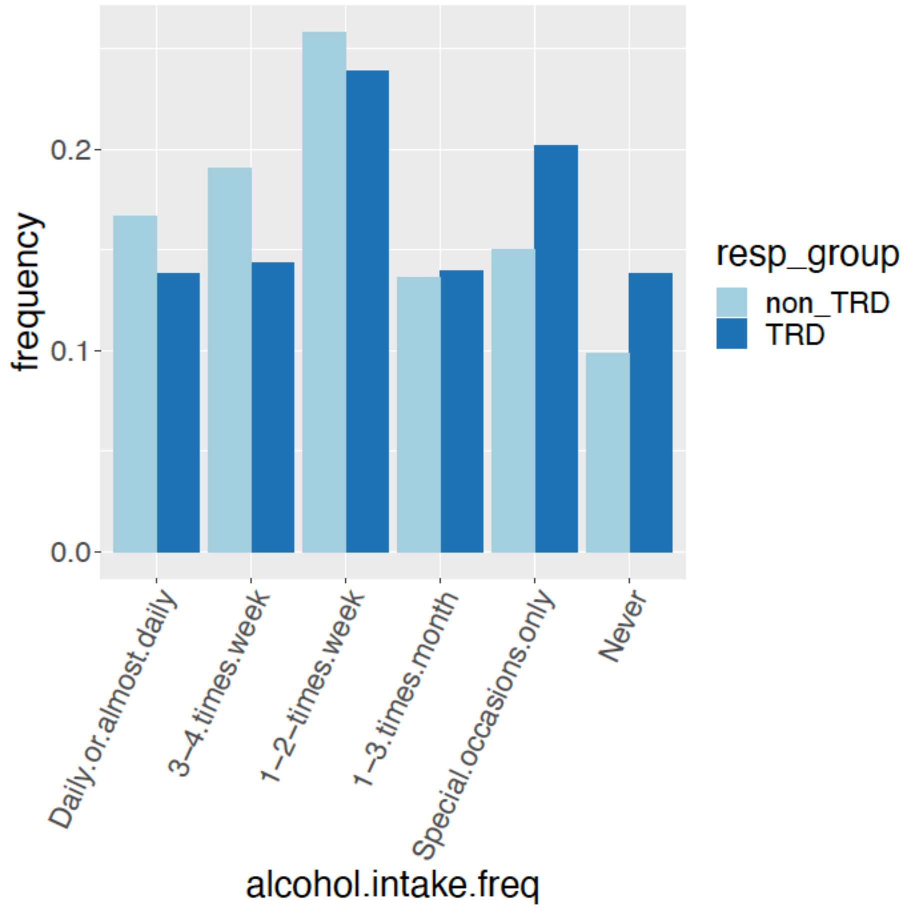

Supplementary Figure 5: frequency of antidepressant (AD) combinations (**A**), antidepressant augmentation with an antipsychotic (AP) (**B**) and a mood stabilizer (MS) (**C**) grouped by drug class between participants with treatment-resistant depression (TRD) and non-TRD in UKB. In TRD and non-TRD groups, the percentage of subjects receiving each combination is reported considering the total number of individuals receiving any antidepressant combination (A), antidepressant-antipsychotic (B) or antidepressant-mood stabilizer (C) combination.

A

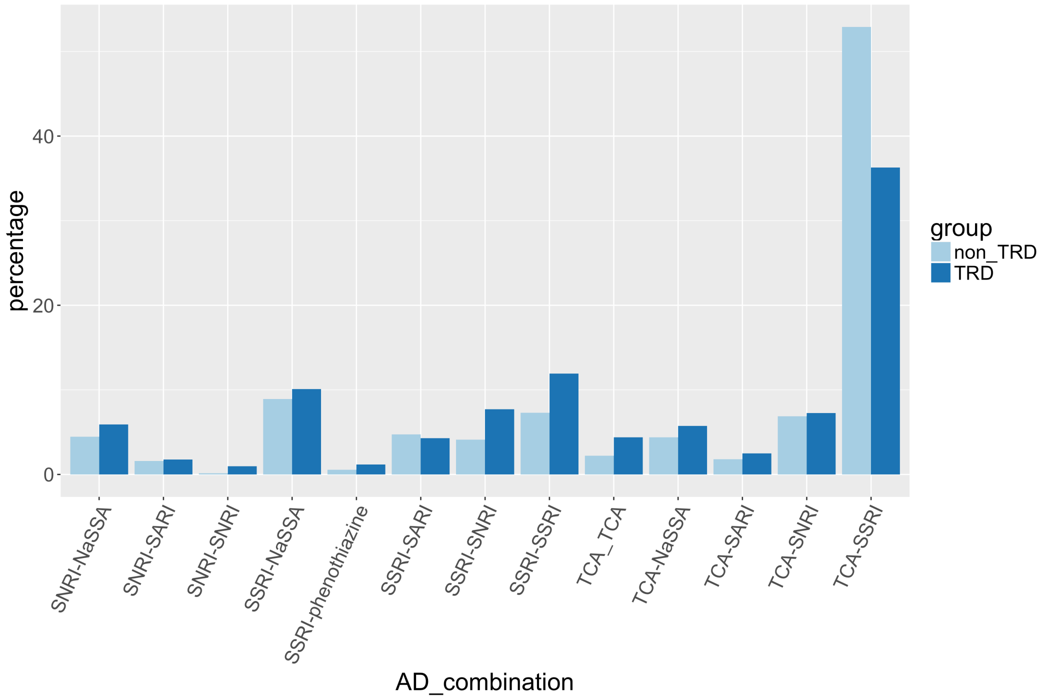

B

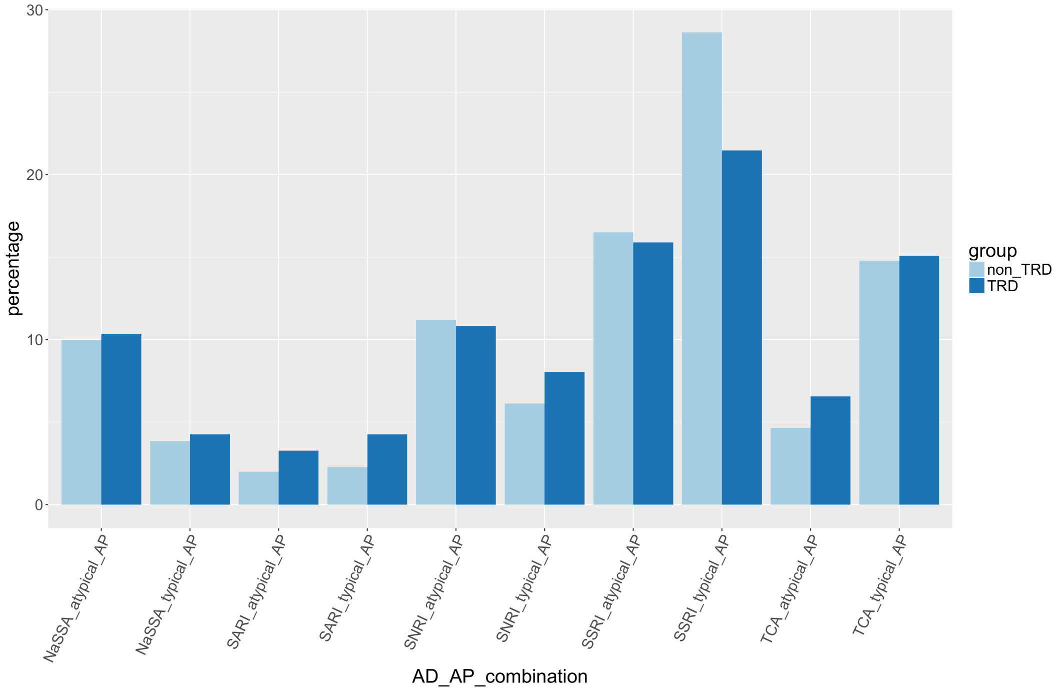

C

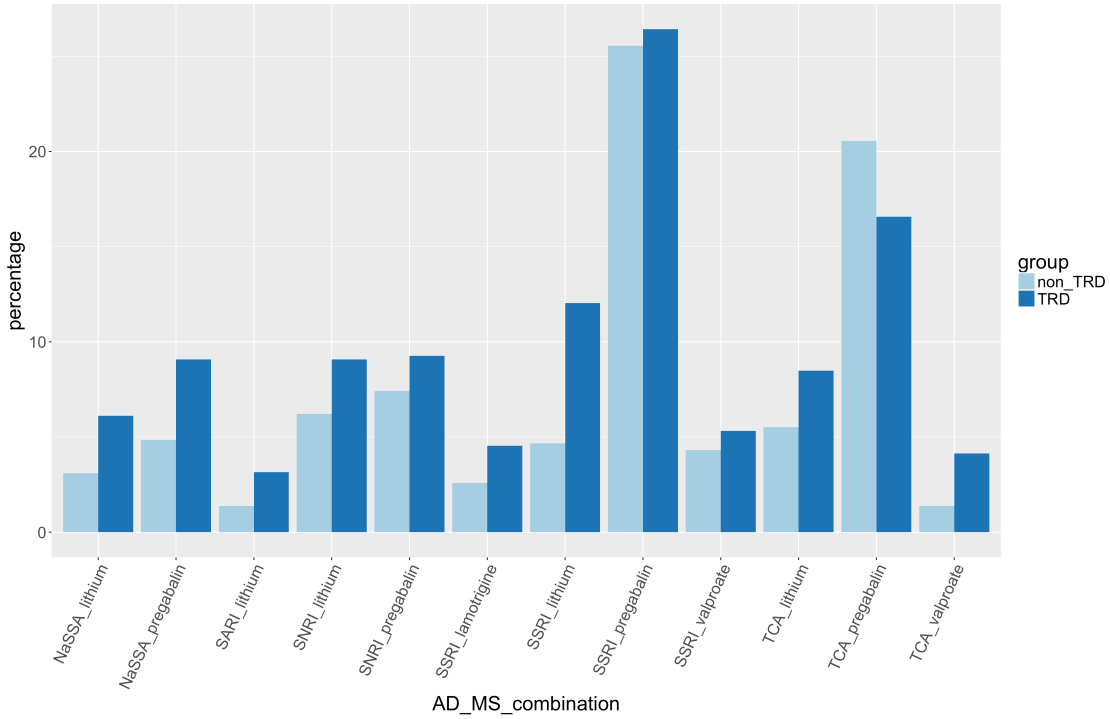

percentage
0
10
20
NaSSA_lithium
NaSSA_pregabalin
SARI_lithium
SNRI_lithium
SNRI_pregabalin
SSRI_lamotrigine
SSRI_lithium
SSRI_pregabalin
SSRI_valproate
TCA_lithium
TCA_pregabalin
TCA_valproate
AD_MS_combination
group
non_TRD
TRD

Supplementary Figure 6: distribution of prescription periods of adequate duration / total prescription periods in each subject split between TRD and non-TRD in UKB. A prescription period was defined adequate when the time between two subsequent antidepressant prescriptions was not longer than 14 weeks.

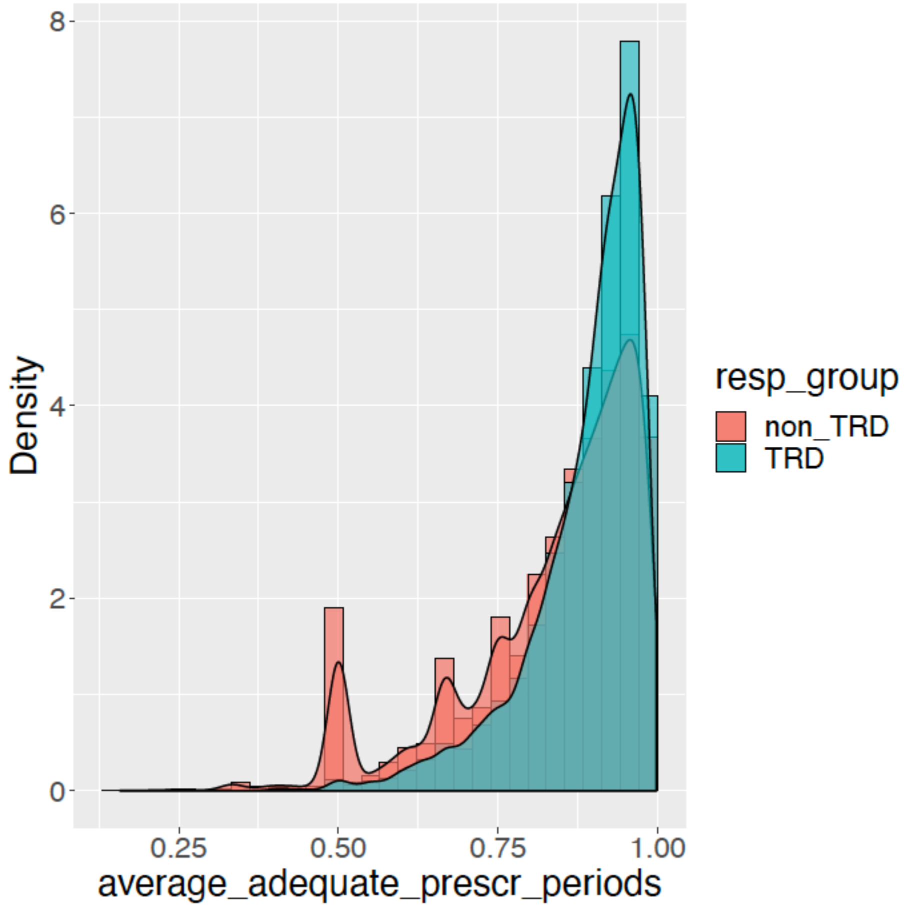

Supplementary Figure 7: distributions of all clinical records (including diagnoses, symptoms, lab results, procedures) and prescription records in TRD and non-TRD groups in UKB (**A**) and EXCEED (**B**). Records from 1985 forwards were considered as previous records were sparse.

A. UKB

Total number of clinical records

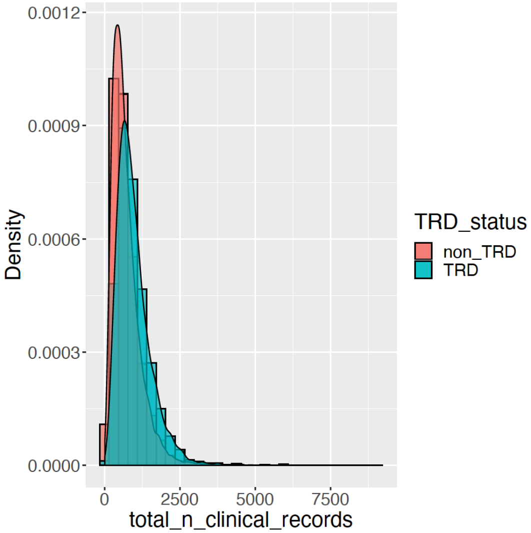

Total number of prescription records

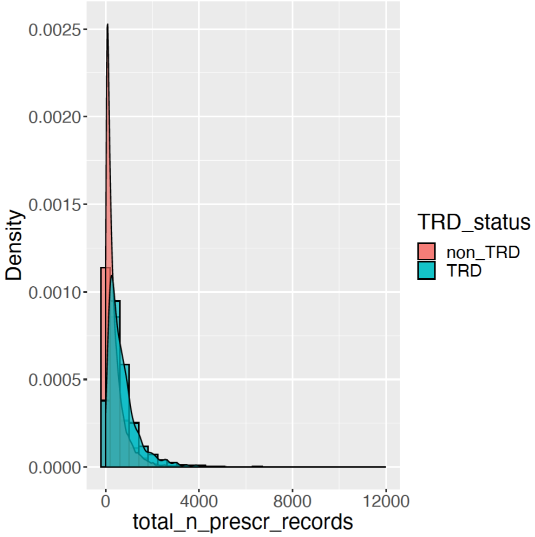

Number of clinical records registered on different dates

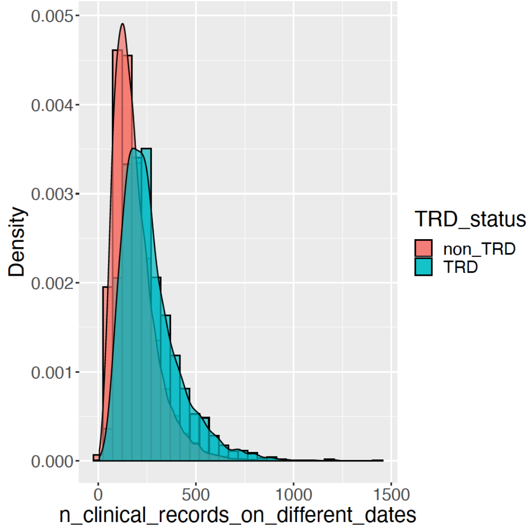

Number of prescription records registered on different dates

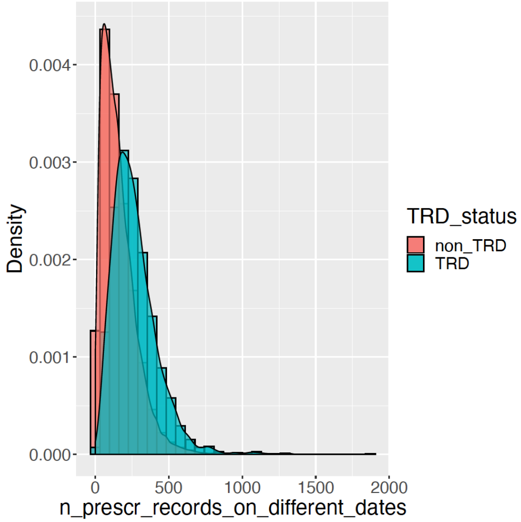

Total number of clinical records / time span of clinical records (years)

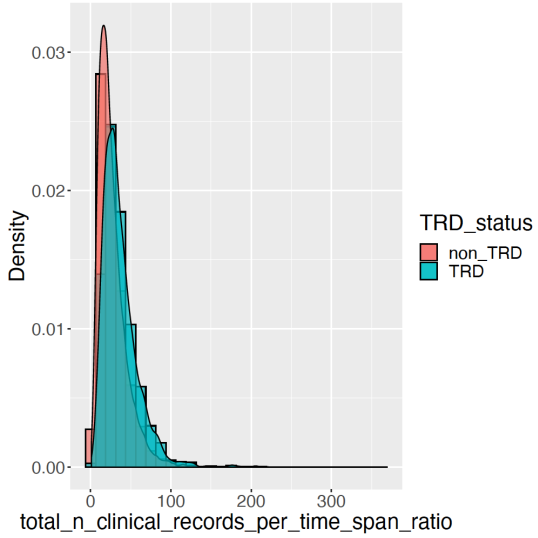

Total number of prescription records / time span of prescription records (years)

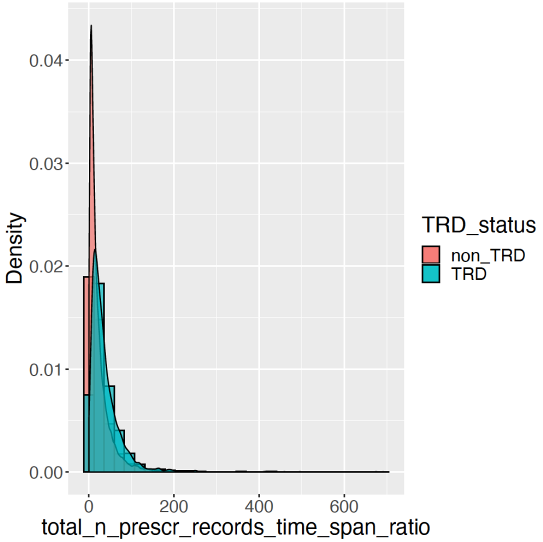

Number of clinical records registered on different dates / time span of clinical records (years)

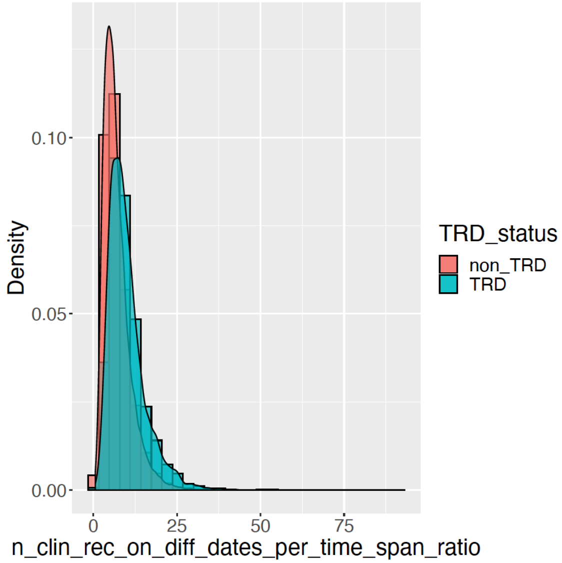

Number of prescription records registered on different dates / time span of prescription records (years)

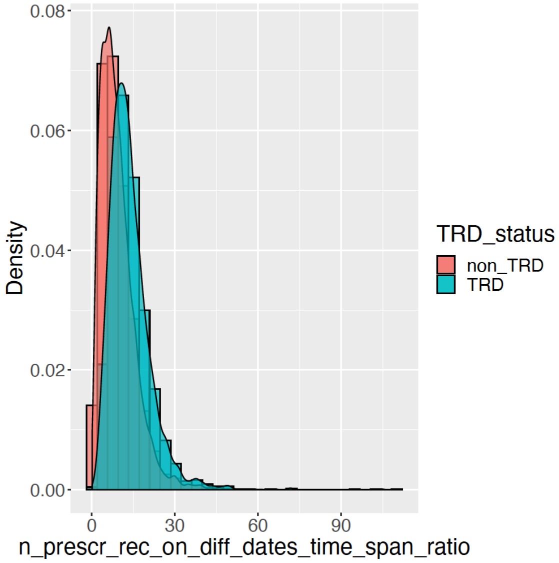

Average time between consecutive clinical records (weeks)

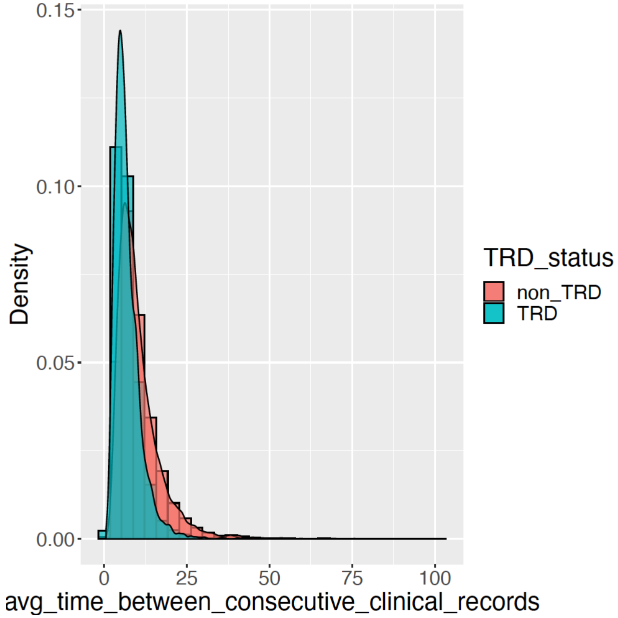

Average time between consecutive prescription records (weeks)

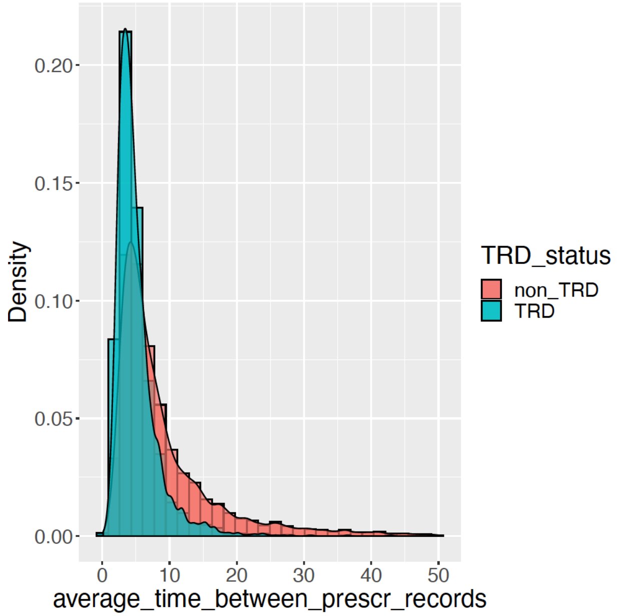

Time span of clinical records (years)

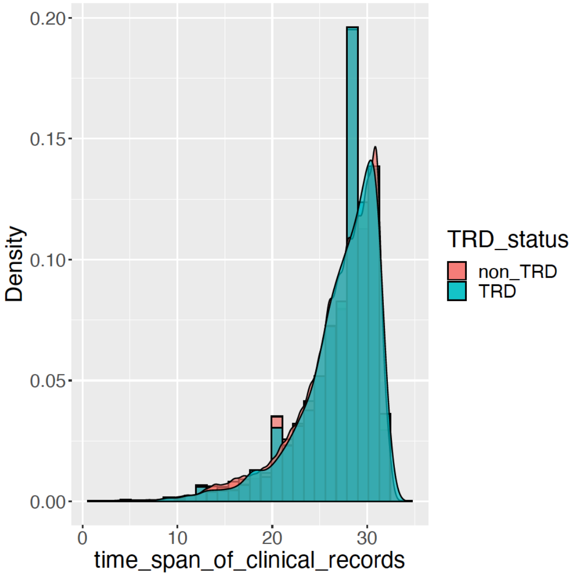

Time span of prescription records (years)

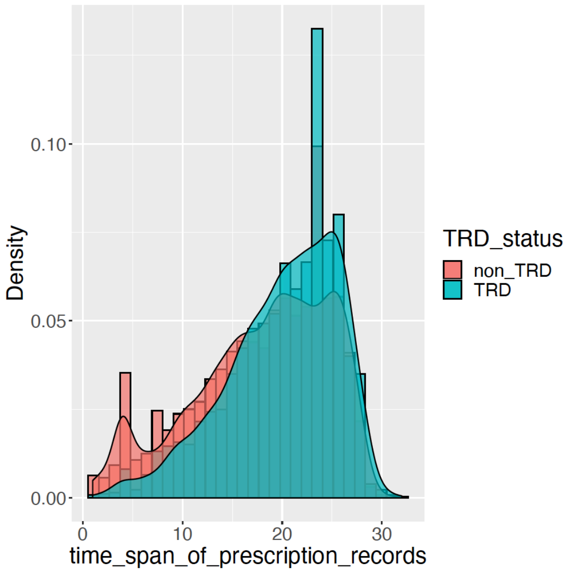

B. EXCEED

Total number of clinical records

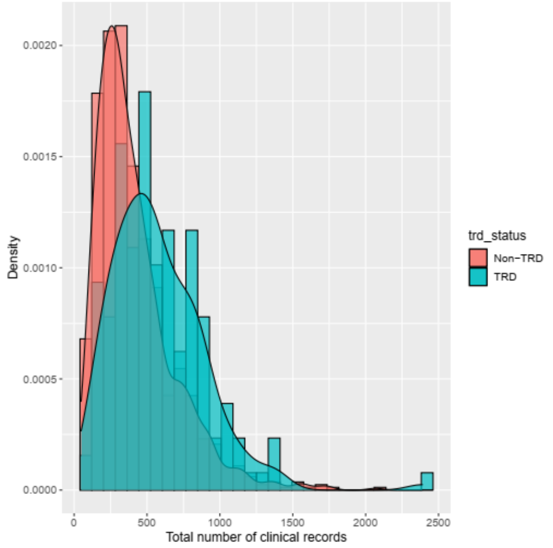

Total number of prescription records

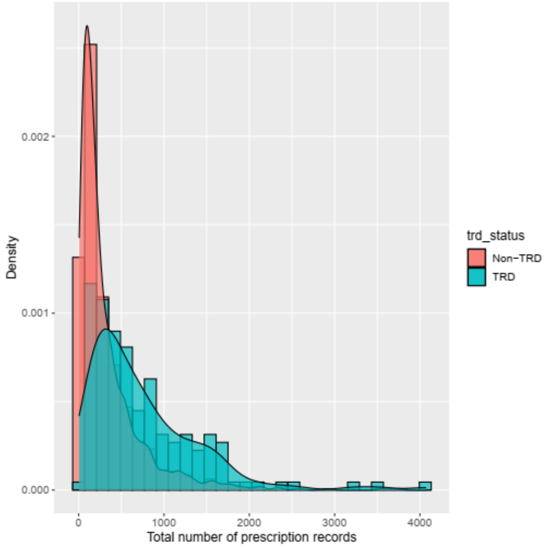

Number of clinical records registered on different dates

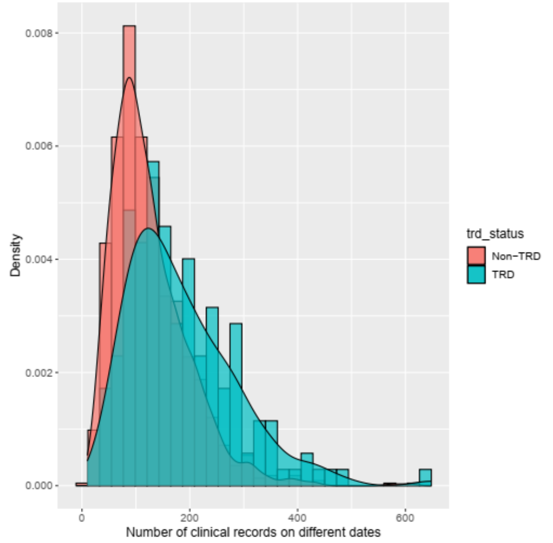

Number of prescription records registered on different dates

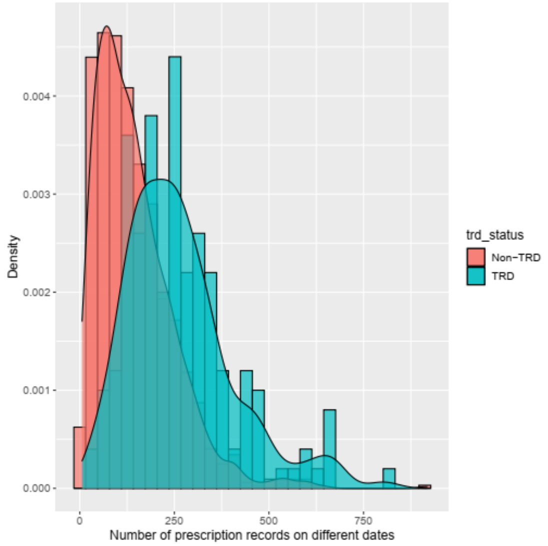

Total number of clinical records / time span of clinical records (years)

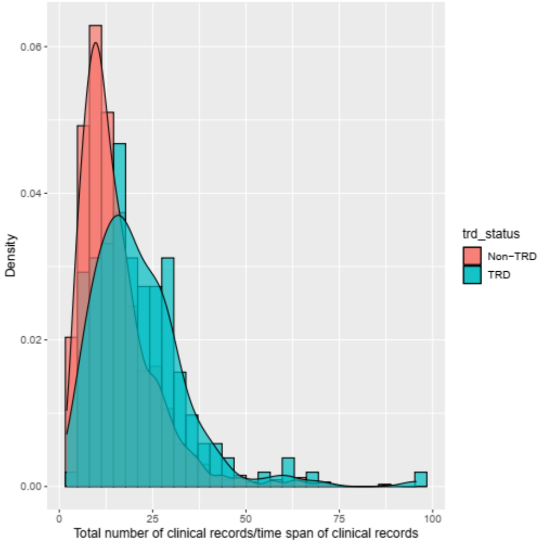

Total number of prescription records / time span of prescription records (years)

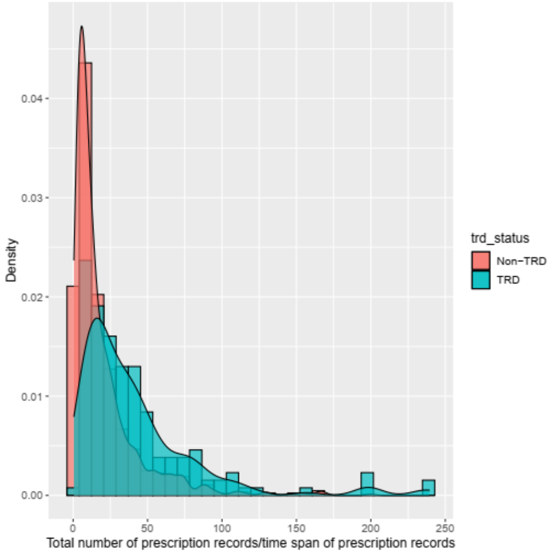

Number of clinical records registered on different dates / time span of clinical records (years)

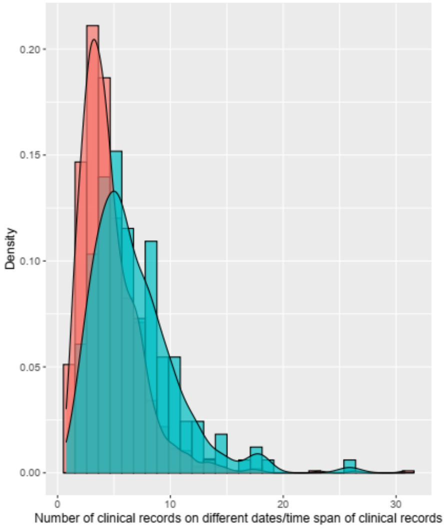

Number of prescription records registered on different dates / time span of prescription records (years)

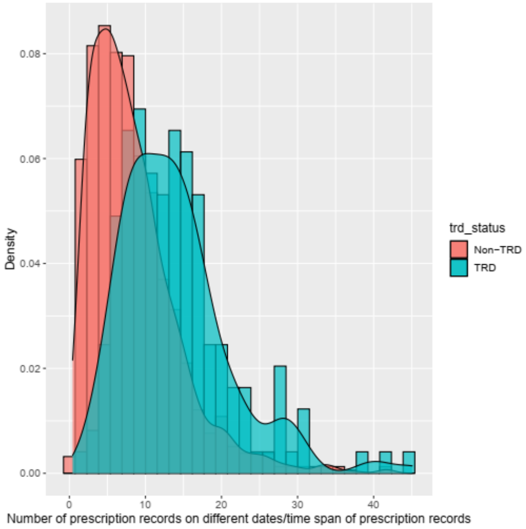

Average time between consecutive clinical records (weeks)

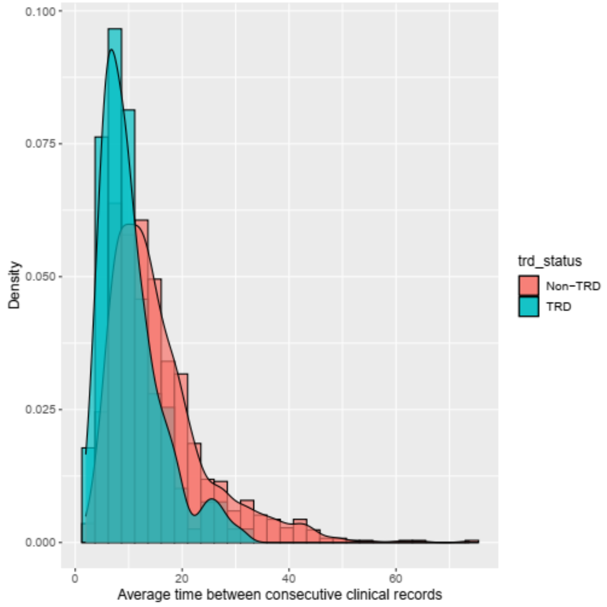

Average time between consecutive prescription records (weeks)

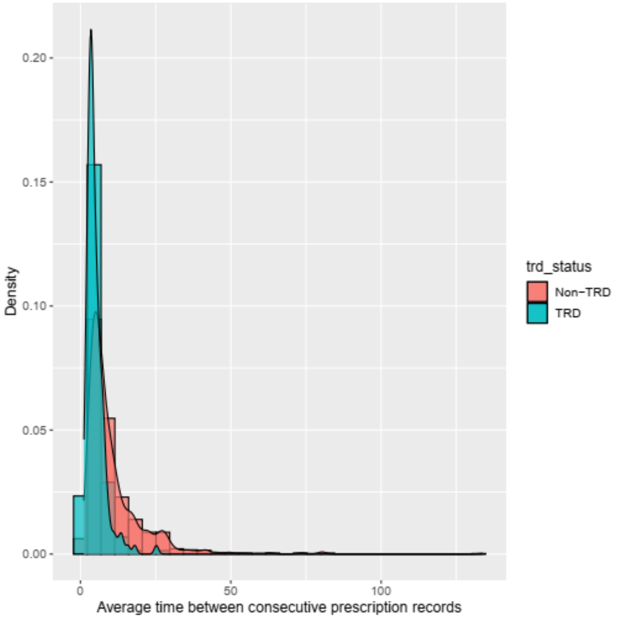

Time span of clinical records (years)

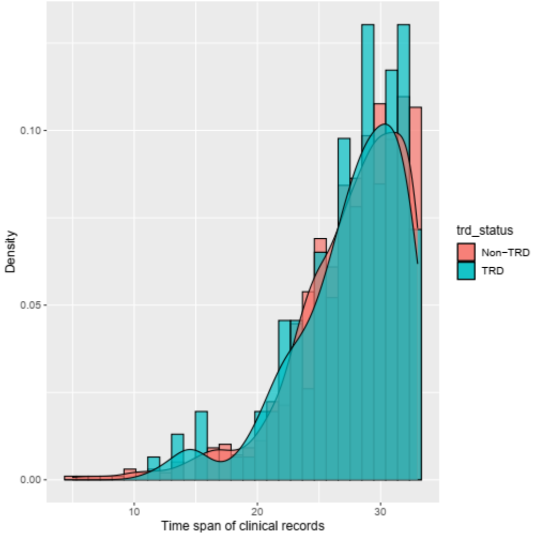

Time span of prescription records (years)

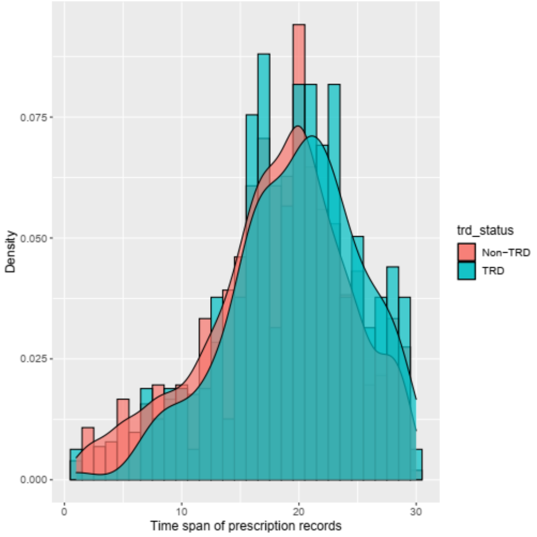

Supplementary Figure 8: genetic correlations of TRD and non-TRD vs. healthy controls (**A**) and TRD vs. non-TRD defined in UKB (**B**) with 30 psychiatric, cognitive, circadian and cardio-metabolic traits.

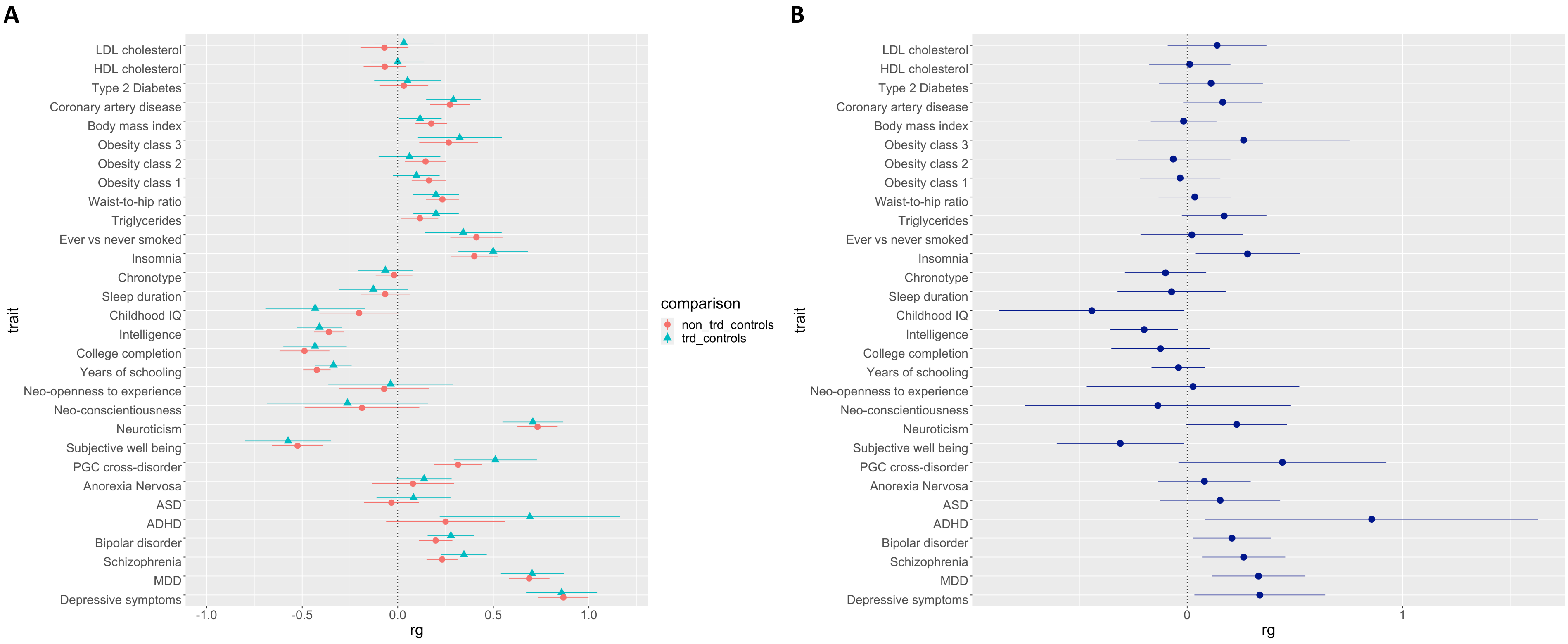

Supplementary Figure 9: PRS results at different p thresholds for attention-deficit hyperactivity disorder in UKB. P-values at the different p thresholds are reported at the top of each column.

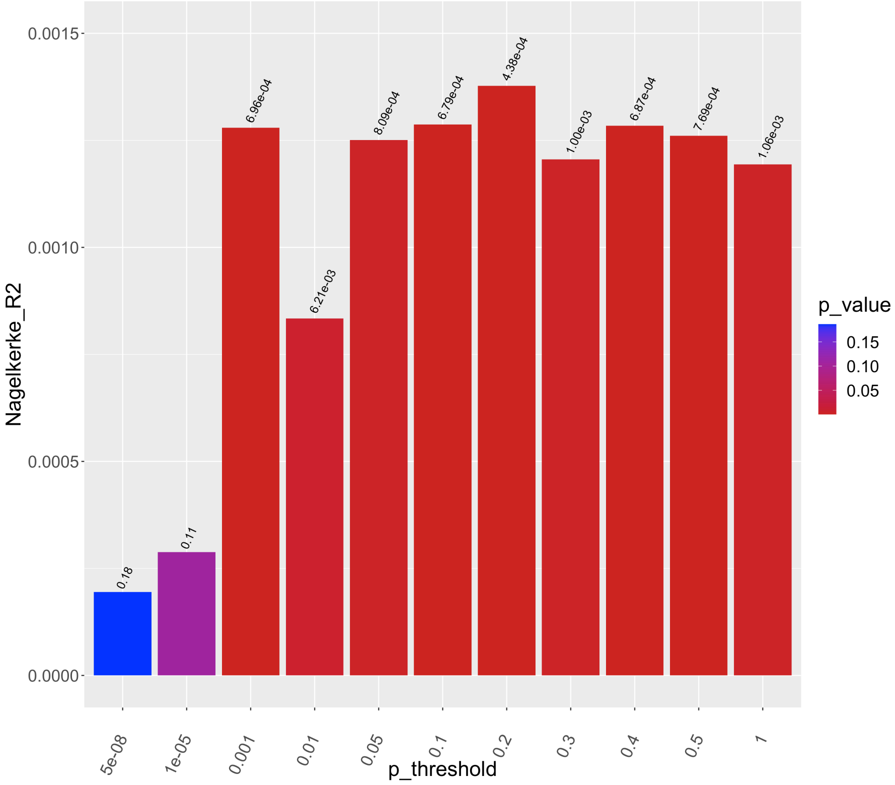

Supplementary Figure 10: OR of PRS and 95% confidence intervals in the prediction of TRD vs. non-TRD in UKB. * p<5.88e-4 (Bonferroni corrected p value).

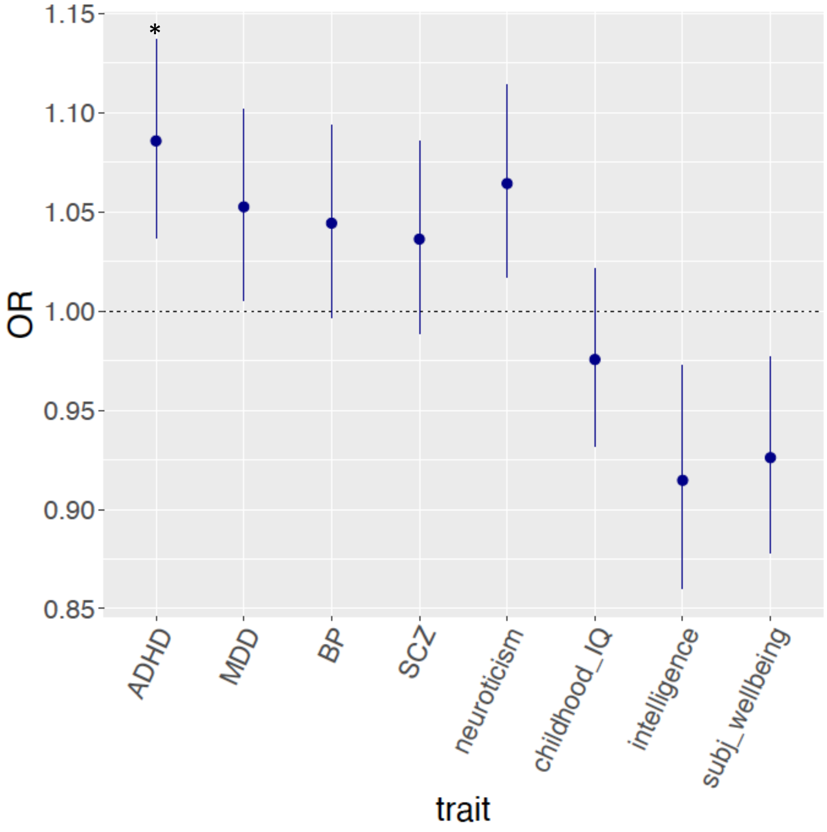

Supplement: Supplementary file 1 — Supplementary Materials [file 41380_2021_1062_MOESM1_ESM.pdf]
